# The adverse role of endocrine disrupting chemicals in the reproductive system

**DOI:** 10.3389/fendo.2023.1324993

**Published:** 2024-01-17

**Authors:** Jing Pan, Pengfei Liu, Xiao Yu, Zhongming Zhang, Jinxing Liu

**Affiliations:** ^1^ The First Clinical College, Shandong University of Traditional Chinese Medicine, Jinan, Shandong, China; ^2^ Gynecology Department, Shandong University of Traditional Chinese Medicine Affiliated Hospital, Jinan, Shandong, China; ^3^ Zhang Zhongjing College of Chinese Medicine, Nanyang Institute of Technology, Nanyang, Henan, China

**Keywords:** endocrine disrupting chemicals, reproductive system, hypothalamic-pituitary-gonadal axis, hormone-like effects, infertility

## Abstract

Reproductive system diseases pose prominent threats to human physical and mental well-being. Besides being influenced by genetic material regulation and changes in lifestyle, the occurrence of these diseases is closely connected to exposure to harmful substances in the environment. Endocrine disrupting chemicals (EDCs), characterized by hormone-like effects, have a wide range of influences on the reproductive system. EDCs are ubiquitous in the natural environment and are present in a wide range of industrial and everyday products. Currently, thousands of chemicals have been reported to exhibit endocrine effects, and this number is likely to increase as the testing for potential EDCs has not been consistently required, and obtaining data has been limited, partly due to the long latency of many diseases. The ability to avoid exposure to EDCs, especially those of artificially synthesized origin, is increasingly challenging. While EDCs can be divided into persistent and non-persistent depending on their degree of degradation, due to the recent uptick in research studies in this area, we have chosen to focus on the research pertaining to the detrimental effects on reproductive health of exposure to several EDCs that are widely encountered in daily life over the past six years, specifically bisphenol A (BPA), phthalates (PAEs), polychlorinated biphenyls (PCBs), parabens, pesticides, heavy metals, and so on. By focusing on the impact of EDCs on the hypothalamic-pituitary-gonadal (HPG) axis, which leads to the occurrence and development of reproductive system diseases, this review aims to provide new insights into the molecular mechanisms of EDCs’ damage to human health and to encourage further in-depth research to clarify the potentially harmful effects of EDC exposure through various other mechanisms. Ultimately, it offers a scientific basis to enhance EDCs risk management, an endeavor of significant scientific and societal importance for safeguarding reproductive health.

## Introduction

Estrogen, an essential class of sex hormones, plays a pivotal role in maintaining normal physiological functions within the human body. It has been demonstrated to have effects on many physiological events, including cardiovascular function, ovarian endocrine and reproductive function, and even male reproductive function. For example, premenopausal women exhibit a lower incidence and severity of hypertension compared to men of the same age. Our previous study established a close relationship between the shortage of estrogen and an increased risk of hypertension, which is closely related to estrogen receptors (1–3). Unlike the shortage of physiological estrogens, there are abundant xenoestrogens from industrial or natural sources in the ambient environment, which have been proved to be detrimental to a variety of physiological processes through estrogen receptors (ERs) and the subsequent cellular and endocrine signaling pathways by mimicking the endogenous ligand. Understanding this complexity is essential for comprehending the effects of endocrine disruptors on reproductive health.

**Figure 1:**
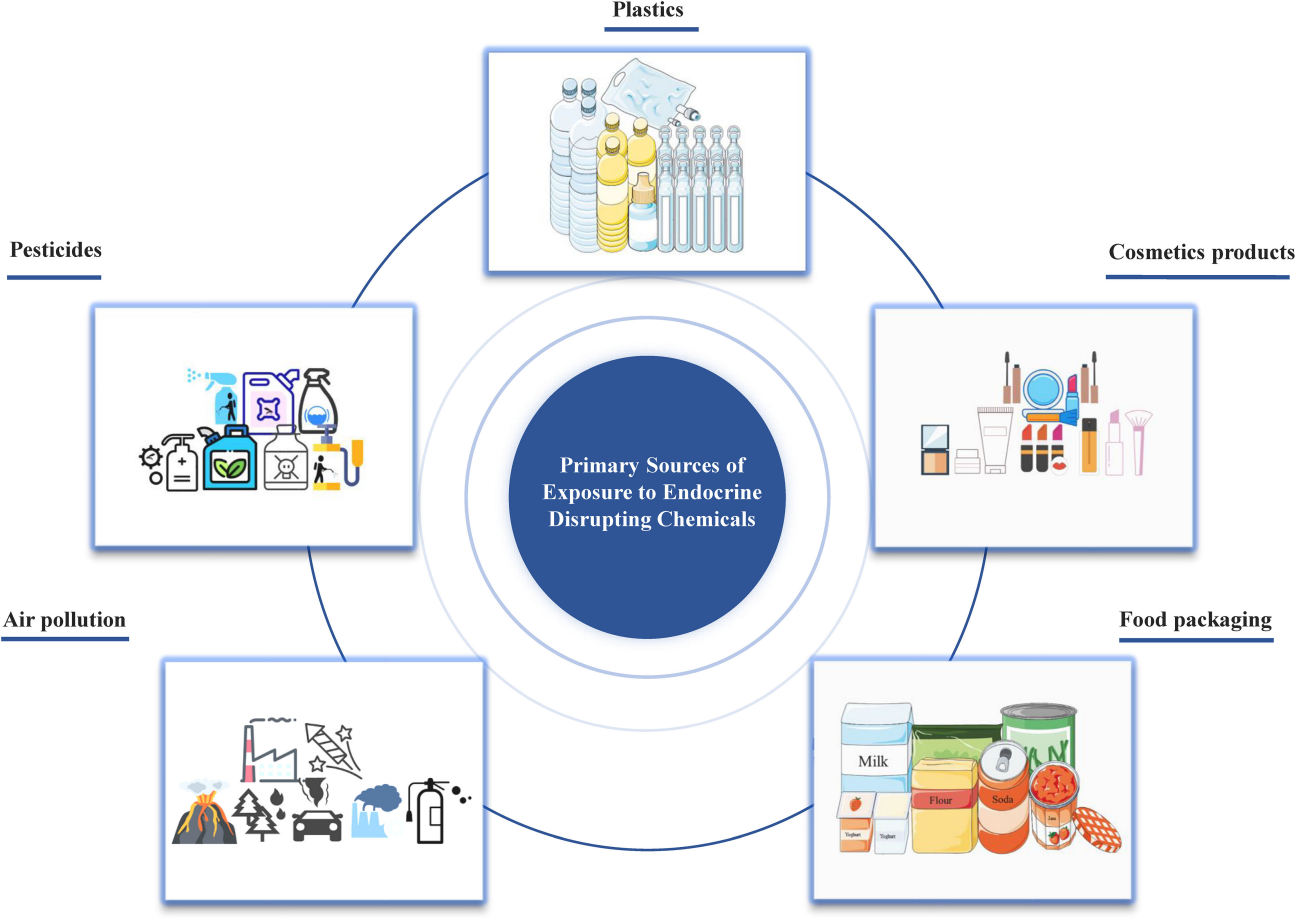
Primary sources of EDCs emission to the environment.

Originally named xenoestrogens, endocrine disrupting chemicals (EDCs) have been officially defined by the USA Environmental Protection Agency (EPA) as external agents that disorder the natural processes involving hormone synthesis, secretion, transport, binding, action, or elimination (4). These chemicals may have estrogenic, anti-estrogenic, androgenic or anti-androgenic effects, which are crucial for maintaining homeostasis, reproductive functions, development, and behavior. The increasing prevalence of human endocrine-related diseases, particularly disorders of the female and male reproductive systems, and even certain cancers (e.g., breast cancer, endometrial cancer, ovarian cancer, cervical cancer, prostate cancer, and testicular cancer), has aroused people’s attention to EDCs (5–7). EDCs represent a global issue of great significance for humans and the environment^8^). With the advancement of science and technology and the development of society, humans are consistently exposed to EDCs through ingestion, inhalation, and skin contact from the embryonic stage, as they are widely present in cosmetics, detergents, pesticides, fungicides, pharmaceuticals, medical equipment, plastics, plasticizers, food products, food and beverage packaging, electronic components, and industrial solvents (**Figure 1**).

As is generally known, EDCs have the capacity to disrupt the normal functioning of the gonads, impacting the regulation of sex hormone biosynthesis controlled by the hypothalamic-pituitary-gonadal (HPG) axis (9, 10). The HPG axis serves as a principal modulator of reproductive function, driving processes such as the growth, development, maturation, and ovulation of follicles, as well as spermatogenesis and steroidogenesis (11, 12). The regulation of the physiological activities of the human reproductive system involves the hypothalamus, pituitary gland, and gonads, which regulate the secretion of gonadotropin-releasing hormone (GnRH), gonadotropins (Gn), and gonadal hormones (e.g., estrogen, progestogen, and androgen). The interactions among these hormones are critical for the proper control of the HPG axis. Most EDCs have diverse structures, including phenolics and non-phenolics of natural or industrial origin, and exhibit hormone-like or anti-hormone-like activities, making the female and male reproductive systems vulnerable to disruption by EDCs. Specifically, EDCs mimic sex hormones and interfere with the release of GnRH and Gn through various pathways, disrupting the HPG axis homeostasis and impairing folliculogenesis, ovulation, conception, spermatogenesis, sperm quality, and reproductive ability. Accordingly, EDCs play an adverse role in the reproductive system, at least in part, by affecting the HPG axis, causing abnormal sensitivity to steroid feedback mechanisms and deleterious effects on female and male reproductive organs and tissues. Further research showed that EDCs not only directly responsible for activating or inhibiting hormonal action but also indirectly modulating their action by autocrine or paracrine signaling, and even signal crosstalk.

Overall, the role of EDCs appears to be quite complicated and includes involvement in the physiology of reproductive organs and tissues (e.g., ovary, breast, endometrium, testis, and prostate), protein synthesis, lipid and bone metabolism, the occurrence and development of ovarian dysfunction, cancer, and other reproductive system-related diseases. Accumulating evidence from animal models and human epidemiological studies highlights the relationship between EDCs and an array of reproductive disorders. These disorders encompass uterine fibroids (UFs), endometriosis (EMs), polycystic ovary syndrome (PCOS), diminished ovarian reserve (DOR), premature ovarian insufficiency (POI), infertility, breast cancer (BC), endometrial cancer (EC), ovarian cancer (OC), cervical cancer (CC), cryptorchidism, hypospadias, prostate cancer (PC) and testicular cancer (TC). EDCs like bisphenol A (BPA), phthalates (PAEs), polychlorinated biphenyls (PCBs), parabens, pesticides, heavy metals, among others, have been implicated in these diseases, highlighting potential reproductive health risks of those EDCs during critical susceptibility periods.

EDCs are now recognized as serious threats to public health and one of the leading environmental risks globally. Given the grand challenges faced in the realm of reproductive health, it is imperative to augment research efforts in this sphere and urgent actions need to be taken to reduce extensive exposure to disruptive EDCs. Intending to inform future research and policy, this article aims to update the evidence supporting previously identified or increasingly likely associations of commonly encountered EDCs with female and male reproductive health over the past six years, which is indexed in PubMed, Scopus, and Web of Science regardless of conference abstracts. The exclusion criteria included all articles that were duplicates, unrelated, inaccessible, and not written in English. A large supplementary table summarizing all studies reviewed that reported significant or epidemiologically meaningful associations can be found in the appendix.

### Research on EDCs in the female reproductive system

EDCs exert estrogenic effects, which is the starting point for studies on the occurrence and progression of endocrine-relevant diseases. Female ovaries serve as the primary gonads responsible for producing and releasing mature eggs, as well as secreting estrogen to regulate endometrial growth and menstrual periodic bleeding. By affecting estrogen receptor activity and interfering with estrogen-related signaling pathways, EDCs alter the estrogen level and feedback regulate the secretion of GnRH and Gn. The perturbation of the hypothalamic-pituitary-ovarian axis can impair the growth and development of eggs and periodic changes in the endometrium, leading to abnormal menstrual cycles, ovulation disorders, and premature ovarian aging, thereby reducing female reproductive ability and causing a series of female reproductive system disorders.

**Figure 2:**
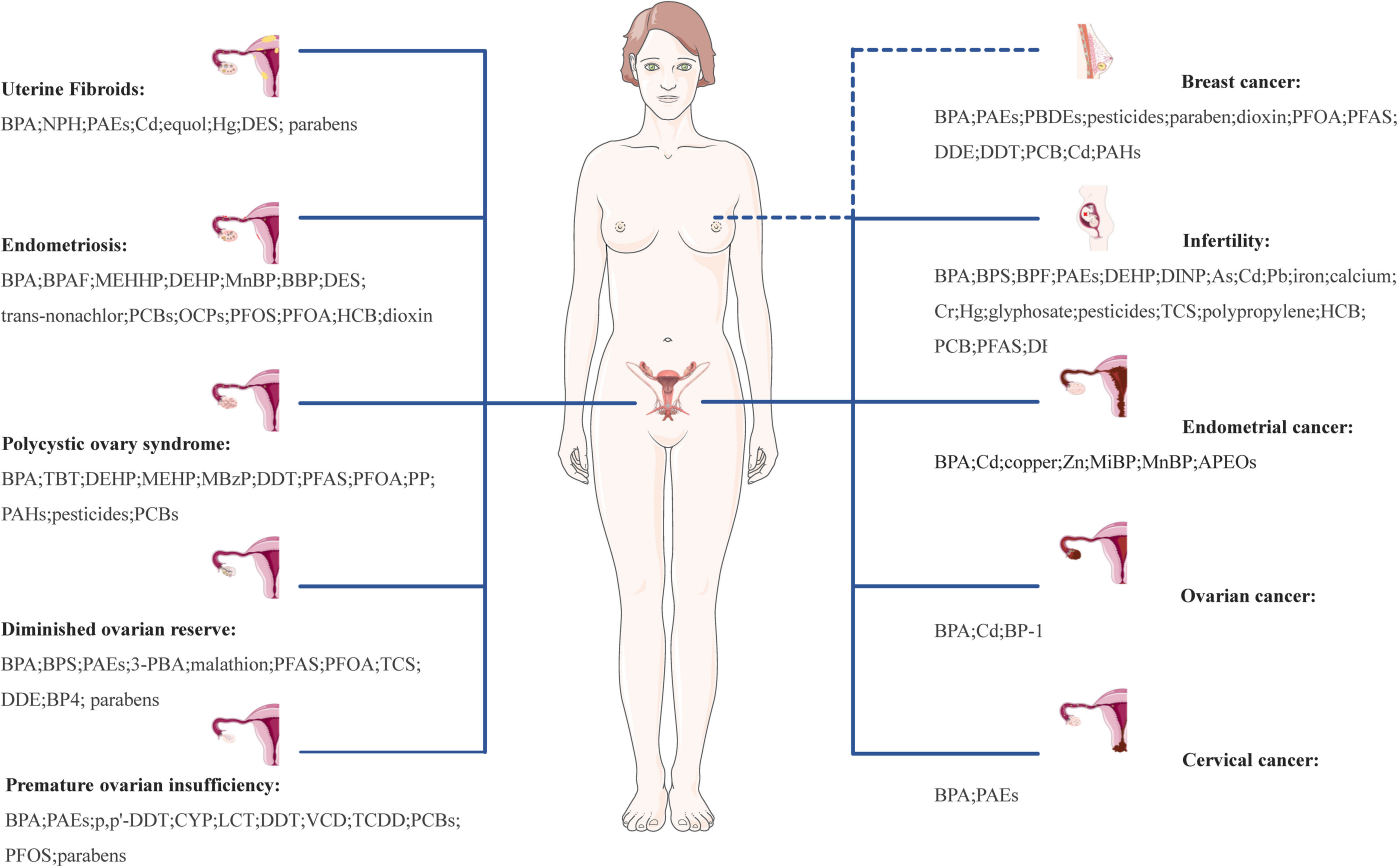
Effect of EDCs on female reproductive health. UFs, uterine fibroids; EMs, endometriosis; PCOS, polycystic ovary syndrome; DOR, diminished ovarian reserve; POI, premature ovarian insufficiency; EC, infertility, endometrial cancer; OC, ovarian cancer; CC, cervical cancer; PC, prostate cancer; TC, testicular cancer; BPA, bisphenol A; NPH, nonylphenol; PAEs, phthalates; Cd, cadmium; Hg, mercury; DES, diethylstilbestrol; BPAF, bisphenol AF; MEHHP, mono-(2-ethyl-5-hydroxyhexyl) phthalate; DEHP, di(2-ethylhexyl)phthalate; MnBP, mono-n-butyl phthalate; BBP, n-butylbenzyl phthalate; PCBs, polychlorinated biphenyls; OCPs, organochlorine pesticides; PFOS, perfluorooctane sulphonate; PFOA, perfluorooctanoic acid; HCB, hexachlorobenzene; TBT, tributyltin; MEHP, mono(2-ethylhexyl) phthalate; MBzP, monobenzyl phthalate; DDT, dichlorodiphenyltrichloroethane; PFAS, perfluorinated substances; PP, propylparaben; PAHs, polycyclic aromatic hydrocarbons; 3-PBA, 3-phenoxybenoic acid; TCS, triclosan; DDE, p,p’-dichlorodiphenyldichloroethylene; BP4, benzophenone-4; CYP, cypermethrin; LCT, lambda-cyhalothrin; VCD, 4-vinylcyclohexenediepoxide; TCDD, 2,3,7,8-tetrachlorodibenzo-p-dioxin; DINP, diisononyl phthalate; As, arsenic; Pb, lead; Cr, hexavalent chromium; Zn, zinc; MiBP, monoisobutyl phthalate; APEOs, alkylphenol ethoxylates; BP-1, benzophenone-1.

Chronic, long-term exposure to EDCs can cumulatively induce hormone-like effects, leading to endocrine dysfunction and adverse effects on the structure and function of female reproductive tissues and organs, potentially contributing to the development of numerous complex diseases, including UFs, EMs, PCOS, DOR, POI, infertility, EC, OC, CC, and BC. A comprehensive overview of common EDCs related to female reproductive system diseases is shown in **Figure 2**.

#### EDCs impact uterine leiomyoma cells’ proliferation ability leading to uterine fibroids (UFs)

Among women in their reproductive age, UFs are the prevailing benign tumors. Their growth is under the influence of sex steroids, leading to their appearance during the reproductive years and subsequent regression after menopause. The growth of fibroids is driven by higher levels of estrogen and progesterone in the body, making them a significant factor in the development of UFs. Conspicuously, exposure to various EDCs commonly found in plastics, cosmetics, and personal care products, including bisphenol A (BPA), phthalates (PAEs), cadmium (Cd), and diethylstilbestrol (DES), has been associated with an elevation in sex hormone levels and the development of UFs in women.

##### BPA

BPA was first synthesized in 1981 and has been utilized in the production of plastics since the 1950s (13, 14). In addition to its role as a constituent of plastics, BPA is commonly found in epoxy resin linings for metal-based food and beverage cans. Furthermore, it is utilized in various consumer products such as thermal paper, medical equipment, toys, electronics, and water pipes (15, 16). It is worth noting that BPA can leach into food, water, or medical supplies through physical manipulation or repetitive use. The United States Environmental Protection Agency (US-EPA) has established a safety level for BPA at 50 μg/kg/d (micrograms per kilogram per day), whereas the European Food Safety Authority (EFSA) lowered its temporary tolerable daily intake to 4 μg/kg/d (17). This demonstrated that BPA is well-known as one of the most infamous EDCs, even at very low concentrations, BPA can also be combined with ERs and activate ER-dependent transcription, resulting in the corresponding biological effect, and causing negative effects on human health.

BPA exerts a significant impact on the development of UFs through environmental exposure and lifestyle factors (18, 19). Experimental studies using human uterine leiomyoma (UL) cells have demonstrated that BPA, at low concentrations ranging from 10^-6^μM to 10μM, enhances cell proliferation by facilitating hormonally regulated progression, while higher concentrations (100 μM-200μM) inhibit growth (20). Further investigations, involving intervention with BPA in primary and subcultures of human UF cells, have provided compelling evidence suggesting that BPA induces dose and time-dependent promotion of leiomyoma cell growth. This effect is achieved through the activation of signaling pathways such as GPR30-EGFR and MAPK/ERK/c-fos (21). Additional investigations have shown that BPA and its analog, nonylphenol (NPH), can activate the TGF-β signaling pathway through estrogen receptor-α (ER-α) signaling, thereby modulating the proliferation of UL cells *in vitro* (22). The application of RNA-seq analysis has revealed that BPA and NPH, acting as external stimulants with phenolic estrogen properties, induce the upregulation of inflammatory factors in uterine leiomyomas (23). By combining ChIP-seq and RNA-seq methods, researchers have gained new insights into the pathogenesis of UFs, specifically how BPA and its derivative can facilitate fibrosis through the PI3K/AKT signaling pathway (24).

Recent research highlights BPA’s significant influence on ER activity, inflammation, and related signaling pathways, which in turn affect the proliferation and apoptosis of UL cells, ultimately promoting fibroid growth and development. However, a study focusing on reproductive-aged Black women did not find consistent and strong associations between urinary BPA concentrations and the incidence or growth of UFs (25). Consequently, in order to comprehensively evaluate the relationship between BPA and UFs, large-scale multi-species research is necessary.

##### PAEs

PAEs, as a plasticizer, are extensively used in certain detergents, medical products, and even as coatings for oral drugs and dietary supplements (26). They are also known to have endocrine-disrupting properties owing to their ability to interfere with the action or metabolism of sex hormones (27). The urinary concentration of PAE metabolites is commonly used as a representative biomarker of exposure to di (2-ethylhexyl) phthalate (DEHP). An in-depth analysis of baseline data obtained from the Midlife Women’s Health Study (MWHS) revealed a strong positive association between higher levels of ΣDEHP and related phthalate molar sums with the risk of prior fibroid diagnosis, particularly in women who experienced weight gain and obesity from early adulthood to midlife (28). Additionally, a study combining questionnaire survey data and high-performance liquid chromatography measurements found higher urine concentrations of DEHP in women with UFs, demonstrating positive associations between DEHP and uterine volume (29). A preliminary study investigating PAEs exposure and the burden of UFs among women undergoing surgical treatment found consistent associations between DEHP metabolites and uterine volume that were independent of race or ethnicity (30). Furthermore, in a mouse xenograft model, human fibroid tissue exposed to DEHP exhibited increased proliferation, tumor growth, and collagen production compared to tissue from animals fed a control substance (31). *In vitro* experiments using human uterine leiomyoma cells demonstrated that DEHP exposure enhanced cell viability, promoted anti-apoptotic protein expression, and induced the expression of HIF-1α and COX-2 (32). Other epidemiological and *in vitro* studies have shown that the major DEHP metabolite, mono (2-ethyl-5-hydroxyhexyl) phthalate (MEHHP), enhances the survival of leiomyoma cells through activation of the tryptophan-kynurenine-AHR pathway (33). Additionally, analysis of the TREE cohort data revealed positive associations between certain PAE exposures and the risks of UFs and endometriosis, which may be mediated by oxidatively generated DNA damage (34). Compared to conventional PAEs, a case-control study among Korean women before menopause conducted organophosphate esters (OPEs) and some alternative plasticizers are associated with increased risks of UFs (35).

However, a prospective ultrasound study showed little evidence of the effect of the PAE biomarkers mixture on uterine leiomyomata incidence (36). It is worth noting that the interplay of racism and sexism may potentially influence the research findings (37). Taking into consideration the simultaneous exposure to chemicals through common sources and metabolic pathways, multivariable logistic regression, WQS regression, and BKMR regression models were employed to investigate the joint effects of ten commonly encountered endocrine-disrupting chemicals, including urinary metabolites of PAEs and mono (2-ethylhexyl) phthalate (MEHP), revealing negative associations with uterine leiomyomas and endometriosis (26).

Although controversies persist, the majority of findings suggest that PAEs and their metabolites may contribute to oxidative stress and inflammatory responses, thereby promoting proliferative and anti-apoptotic activities in human uterine leiomyoma cells.

##### Other EDCs

In addition to the detrimental effects of BPA and PAEs on UFs development, analyses using simple and multiple linear regression revealed that elevated blood cadmium (Cd) levels were positively correlated with UF development in a group of 308 premenopausal women aged 30-49 years (38). Notably, a study conducted on fibroid cells indicated that exposure to Cd-induced changes in gene expression, leading to increased cellular movement and invasiveness (39). Furthermore, continuous exposure to Cd resulted in the transformation of benign human UL cells into malignant cells *in vitro*, which was accompanied by the downregulation of genes encoding for extracellular matrix components and the upregulation of genes involved in ECM degradation (40). Mechanistic research revealed that Cd-induced proliferation of human UL cells occurred through activation of Histone H3 and Aurora B via Fork head box M1/Cyclin D1 interactions downstream of MAPK and a nongenomic GPER/p-src/EGFR/MAPK signaling pathway that did not directly involve ERα (41, 42). Another noteworthy EDCs, diethylstilbestrol (DES), a synthetic nonsteroidal estrogen once used to support pregnancy and prevent and treat osteoporosis could lead to various uteropathies (43). A nationwide retrospective observational study on 529 families of DES-treated women showed that DES was associated with a higher incidence of UFs in women and highlighted a multigenerational and likely transgenerational effect of this EDC in humans (44). Female Eker rats exposed to DES 11μg/d on days 10, 11, and 12 after birth reprogramed the methylome and found an augmenting estrogen activity of myometrial stem cells, leading to a “hyper-inflammatory phenotype” and an increased hormone-dependent risk of UFs (45, 46). Further investigation into the molecular mechanisms implicated that early life exposure to DES suppressed nucleotide excision repair in rat myometrial stem cells through TGF-β signaling (47). Moreover, studies demonstrated that Vitamin D3 could mitigate the DNA damage caused by developmental DES exposure in uterine myometrial stem cells, effectively reducing the incidence of UF development (48). In comprehensive statistical analyses aiming to explore the associations between chemical mixtures and gynecological disorders, equol and mercury (Hg) were identified as the most significant chemicals linked to uterine leiomyomata (49, 50). In one study of reproductive-aged Black women, urinary concentrations of some phenols and parabens were weakly and non-monotonically associated with negative UFs growth (25). Parabens are commonly utilized as preservatives in various foods, including sauces, jams, and soft drinks, as well as in cosmetic products (51). Research in rodents has demonstrated that parabens can disrupt ovarian function and reduce fertility (52). It is biologically plausible that parabens act in an anti-estrogenic manner in the uterus, which could explain the finding of a weak inverse association between paraben exposure biomarkers and UFs incidence.

*In vitro* and *in vivo* studies have shown that BPA, NPH, DEHP, Cd, DES, Hg, and equol can cause DNA damage or impact epigenetic processes to fibroid pathogenesis, and early adolescence may be a window of susceptibility for fibroid development (53). These findings hold promise for the development of new prevention strategies for fibroids. However, further research at more realistic exposure levels is necessary to identify critical biological pathways and accurately assess the risks associated with windows of exposure to the aforementioned EDCs, taking into consideration underlying mechanisms and species-specific differences.

#### EDCs enhance invasive and proliferative activities of endometrial cells in endometriosis (EMs)

EMs, being a hormone-dependent chronic inflammatory disease, has a multifactorial etiology involving genetic, hormonal, immunologic, and environmental factors. Characterized by the implantation and growth of endometrial tissue outside the uterine cavity, EMs are strongly associated with high estrogen secretion. Numerous studies have indicated a potential link between exposure to EDCs, particularly BPA and PAEs, and abnormal secretion of estrogen in the HPG axis, thereby playing a role in the etiology of EMs (54–58). Nevertheless, further research is required to systematically evaluate the probability and strength of these exposure-outcome relationships.

##### BPA

Exposure to BPA increased the odds of having EMs in a dose-response relationship (59), and oxidative stress perhaps plays a potential role in the endocrine-disruptive (60). Histopathological examinations of the uterus in CD1 mice exposed to varying doses of BPA revealed an accumulation of collagen and an elevated number of F4/80 positive macrophages, resembling characteristics associated with an EMs-like phenotype (61). Similarly, experiments using a mouse model of EMs demonstrated that BPA exposure led to an upregulation of ER-β expression and an increase in both the size and number of endometriotic lesions. Co-administration of an ER-β antagonist blunted the effects of BPA on lesion number and volume, suggesting that BPA promotes endometriotic lesion formation and growth in an ERβ-dependent manner. Molecular mechanism studies have demonstrated that BPA modulates the WDR5/TET2 complex, resulting in the regulation of ER-β expression in eutopic endometrium and driving the development of EMs (62). Additionally, BPA treatment of endometriosis stromal cells (ESCs) under serum-free revealed that ESC proliferation was most pronounced upon exposure to 1000 pmol BPA (63). BPA was also found to enhance the invasion of human ESCs by upregulating the expressions of matrix metalloproteinase-2 (MMP-2) and MMP-9 through the GPER-mediated MAPK/ERK signaling pathway, thereby increasing the risk of peritoneal EMs (64). Furthermore, bisphenol AF (BPAF) has been found to disrupt normal ovarian signaling to a greater extent than BPA when administered through the diet in a mouse model of EMs, resulting in increased growth of EMs lesions (65).

Moreover, evidence suggests that BPA and its substitutes may have consequences across generations, compromising the ovarian function of subsequent cohorts. These previous studies confirm the increased incidence of EMs-like lesions associated with BPA exposure, highlighting the importance of conducting further epidemiological studies to evaluate the potential association between BPA and EMs using biomarker analysis.

##### PAEs

The results of the systematic review as of July 2018 revealed PAEs were positively associated with the prevalence of EMs (66). In Asia, research has demonstrated that exposure to MEHHP, a metabolite of PAEs, may pose a potential risk for women with EMs (67), similar to how DEHP affects women’s reproductive prognosis and ovarian function (68). Endometriotic tissue is featured by high prostaglandin levels and progesterone resistance. Microarray studies showed that DEHP upregulated aldo-keto reductases expression in EMs patients and enhanced progesterone resistance (69). Furthermore, treatment of endometrial cells with DEHP may increase extracellular signal-regulated kinase phosphorylation and regulate Pak4 expression collaborate with the increase in migration and invasion of endometrial cells, and therefore with the development of EMs (70). The significant increase in endometrial stromal cells invasiveness observed after DEHP exposure could be a link between DEHP exposure and increased EMs likelihood (71). Additionally, nonobese diabetic/severe combined immunodeficiency mice fed with DEHP have exhibited greater growth and proliferation of endometrial implants compared to those fed with a vehicle (70). PAEs are also suspected of promoting EMs through mechanisms other than binding to estradiol receptors (72). Exposure to mono-n-butyl phthalate (MnBP), a PAE metabolite, in Women with EMs, had been found to potentially affect the health and steroidogenesis of human granulosa cells (73). Chronic exposure to low-dose n-butyl benzyl phthalate (BBP), a widely used plasticizer, has been found to have weak estrogenic activity and may potentially promote the survival of endometriotic tissue through CD44-expressing plasmacytoid dendritic cells (74). This highlights the critical need for policy and regulatory initiatives aimed at identifying and controlling the health-related impacts of plastics. Simultaneously, it underscores the necessity for well-designed cohort studies with significant sample sizes to provide further insights.

*In vitro* results have indicated the capacity of PAEs to induce EMs. However, no meaningful relationship was found between the concentration of BPA metabolites in urinary samples and EMs, as determined through biomarker analysis. Likewise, similar results were derived from the study on PAEs (75). Given the weak strength of the results, additional research is warranted to unravel the mechanisms underlying the interaction between PAEs and EMs.

##### Other EDCs

The risk of developing EMs in adult life was significantly increased by exposure to DES *in utero* (76). A case report noted that all daughters and subsequent granddaughters who were prenatally exposed to DES presented with EMs, suggesting a role of DES in the pathogenesis of this disease (77). Animal studies have shown that DES induces erratic estrous cycles and abnormal hormone levels, demonstrating its efficacy in inducing EMs and suggesting its potential as an alternative approach in rat models (78). Integrating metabolic and cytokine profiling, a comprehensive approach has provided support for the association between EMs and certain persistent organic pollutants (POPs) like trans-nonachlor, polychlorinated biphenyls (PCBs) 114 and dioxin-like toxic equivalents from PCBs (79). PCBs are a halogenated aromatic group of ubiquitous, persistent environmental pollutants, and they can be present in high concentrations in fatty foods such as meat, fish and dairy products (80). Findings from the ENDO Study indicated an association between adipose-to-serum ratio and adipose estrogenic PCB and organochlorine pesticides (OCPs) mixtures and incident EMs (81). OCPs are synthetic pesticides that have been widely used in agricultural programs and epidemics in the past decades. Owing to the multiple toxicities and environmental persistence, OCPs have been banned or restricted in many countries. Higher levels of perfluorooctane sulphonate (PFOS) and perfluorooctanoic acid (PFOA) were observed in EMs patients, as measured in women aged 20-50 years from the National Health and Nutrition Examination Survey (82). Experimental evidence from research on human endometrial stromal cells and endometrial stromal cells indicates that hexachlorobenzene (HCB) induces cell migration and invasion, and enhances aromatase expression levels (83). Female rats exposed to varying doses of HCB for 30 days exhibited enhanced growth of endometriotic lesions and associated abnormal changes characteristic of EMs (84). *In vivo* mouse model confirmed that dioxins and dioxin-like PCBs promoted the development of EMs via enhancing 17β-estradiol biosynthesis and inflammatory response mediated by aryl hydrocarbon (85, 86). Dioxin, a byproduct of combustion and various industrial production, has irreversible “teratogenic, carcinogenic, and mutagenic” toxicity. Human can be exposed to dioxins and their by-products by consuming high-fat foods such as meat, poultry, milk, egg. However, one research analyzed plasma levels of PCBs and did not find any clear association between exposure to PCBs and the risk of EMs (87).

Several *in vitro* studies have demonstrated that EDCs, represented by BPA, PAE and DES, could cause inflammation, invasion, change in cytokines, increased oxidative stress, viability, resistance to hydrogen peroxide, and proliferation of endometrial cells. It is important to note that the mechanism of EDC exposure in relation to EMs is still not fully understood, mainly due to the complex pathophysiology of this gynecological disease. These findings underscore the importance of considering mixtures when assessing exposure-disease relationships. Consequently, further large-scale and homogeneous studies are needed to focus on the interactions and to draw conclusions about the influence of these EDCs on the development of EMs.

#### EDCs promote the characteristic performance of polycystic ovary syndrome (PCOS)

PCOS is a heterogeneous disease characterized by hyperandrogenism, ovulatory dysfunction, and polycystic ovarian morphology, ultimately causing anovulatory infertility in women. The etiology of PCOS has not been elucidated, which may be due to the interaction between specific genetic and environmental factors. Extensive evidence has shown that EDCs can provoke abnormalities in the HPG axis, even at low concentrations, thereby leading to metabolic disorders and suggesting a possible etiological mechanism underlying PCOS (88, 89).

##### BPA

Evidence from numerous studies conducted on infertile women supports a strong association between BPA and PCOS. A meta-analysis showed that PCOS patients had significantly higher BPA levels than those of control groups (90). In fact, as much as 48.28% of PCOS patients were found to have detectable BPA in their urine samples. Furthermore, subsequent investigations revealed a positive correlation between BPA and testosterone levels, serum insulin levels, and the incidence of insulin resistance in PCOS patients (91). Moreover, distinct patterns in the steroid hormone biosynthesis and metabolism of linoleic acid, linolenic acid, and sphingolipids were observed in the blood plasma of BPA-exposed patients, providing valuable insights into the underlying metabolic disorders in PCOS (92). Animal experiments studying molecular mechanisms of action provided insights on the effects of BPA exposure on ovulatory, hormonal, mitochondrial dysfunction, and senescence all hallmarks of PCOS phenotype (93). Additionally, female rats subjected to BPA, tributyltin (TBT), or a combination of both from postnatal day 1-16 exhibited irregular estrus cycles, reduced corpora lutea and antral follicles, and an increased occurrence of atretic follicles and cysts (94). Collectively, the aforementioned reports collectively illustrate the detrimental role of BPA in the development and pathogenesis of PCOS. The molecular effect of BPA on PCOS can be summarized as altering ovarian steroidogenesis (95), exacerbating the state of hyperandrogenism, affecting oocyte development, folliculogenesis, and aggravating metabolic parameters. A study on a single generation revealed the combined effects of parental obesity and transgenerational reproductive toxicity of BPA (96). More intricate research has indicated that ancestral exposure to BPA can induce PCOS-like phenotypes in subsequent unexposed generations by activating cancerous pathways, altering arginine-proline metabolism, insulin signaling, AMPK, HOTAIR regulatory pathways, as well as modulating the upstream regulators ESR1 and TGF signaling in the ovary (97). Moreover, a randomized controlled experiment found higher serum levels of BPA replacement chemicals in women with PCOS (98, which is supported by similar findings in a case-control study (99).

Due to the inherent challenges in fully aligning human and animal study results, it remains difficult to ascertain the exact role of BPA and its replacement chemicals in the pathogenesis of PCOS. Consequently, it is imperative to conduct prospective-designed studies focusing on the early origins of PCOS to shed light on the potential health risks associated with BPA exposure in women with this condition.

##### PAEs

Evidence from clinical research confirms that women with PCOS have higher levels of DEHP in their follicular fluid (FF) compared to control subjects, potentially contributing to pregnancy loss following *in vitro* fertilization (IVF) (100). A nested case-control study focusing on women undergoing IVF provides further support for the role of DEHP in PCOS pathogenesis, as urinary metabolites of PAE metabolites were found to be related to PCOS development (101). It is noteworthy that concentrations of PAE metabolites may also play a role in obesity, glucose, and lipid impairment in women with PCOS (102). The TREE cohort study specifically investigated the individual and joint associations of urinary PAE metabolites with PCOS, indicating that mono (2-ethylhexyl) phthalate (MEHP), monobenzyl phthalate (MBzP), and the overall sum of DEHP were associated with a higher prevalence of PCOS (103). Furthermore, PAEs were found to be linked to metabolic disturbances, including insulin resistance indices and serum triglycerides, in adolescents with PCOS (104). However, a study evaluating DEHP and MEHP levels in adolescents found no significant differences between those with and without PCOS (105).

Considering that PCOS is a complex endocrine and metabolic disorder encompassing reproductive and psychological dysfunctions, exposure to PAEs approximately contributes to PCOS progression through metabolic disruption. Nonetheless, given the existence of contradictory research findings, further investigation is required to identify the complete underlying mechanism of the characteristic performance of PCOS.

##### Other EDCs

Polycystic ovaries, chronic anovulation, and hyperandrogenism are classic features of PCOS. Gas Chromatography Mass-Mass Spectrometer analysis revealed that dichlorodiphenyltrichloroethane (DDT), an organochlorine pesticide, may play a critical role in PCOS pathogenesis by modulating reproductive hormone levels involved in androgen catabolism (106). Exposure to tributyltin (TBT), an organometallic xenobiotic, has been shown to induce reproductive, metabolic, and cardiovascular abnormalities similar to PCOS (107), making it a useful tool in the creation of PCOS rat models (108). The relationship between PCOS and exposure to perfluorinated substances (PFAS) has yielded contradictory findings, with some studies reporting a positive association (109, 110), while others report contradictory findings (111). PFAS is a kind of hard to degradation of synthetic chemicals that can mimic fatty acid and bio-accumulate in adipose tissues, activate the peroxisome proliferator-activated receptor and other pathways to play an endocrine role. Exposure to contaminated food, water, and soil can cause reproductive and developmental toxicity of PFAS to humans (112–114). Epidemiological research has demonstrated elevated levels of PFOA, a PFAS member, in the serum of women with PCOS, which were associated with irregular menstrual cycles (115). Experimental interventions involving PFOA in female mice have shown to be ovotoxic (116). Propylparaben (PP), an antimicrobial preservative commonly used in cosmetics, personal care products, and pharmaceuticals, has been shown to exert detrimental effects on ovarian estradiol secretion and ovulation in both mice and human ovarian granulosa tumor-derived cell lines (KGN) (117). In addition, polycyclic aromatic hydrocarbons (PAHs) (118), pesticides (119), and PCBs (120) are some of the EDCs detected in various matrices such as the serum, plasma, FF, and urine of PCOS patients.

Therefore, EDCs discussed above have the potential to affect hormonal function, ultimately affecting the function of sexual steroids, disrupting metabolic processes, compromising reproductive health, and exacerbating the risk associated with PCOS pathogenesis. Large-scale, double-blind, placebo-controlled randomized clinical trials should be conducted to confirm the findings from the aforementioned studies and gain a better understanding of the underlying mechanisms of action.

#### EDCs reduce the number of follicles causing diminished ovarian reserve (DOR)

The decline in ovarian reserve function represents the initial phase of ovarian aging or failure, which can be attributed to a diminished number of recruitable follicles in the ovaries or a decline in the fertilization capacity of oocytes. The ovary plays a significant role in feedback regulation of the HPG axis and female fertility by providing the eggs and sex steroid hormones for fertilization and the maintenance of reproductive function. Environmental factors have been recognized as significant contributors to the diminishment of ovarian resesrve and reproductive damage (19).

##### BPA

According to clinical practice, the decline in ovarian reserves can be assessed using the antral follicle count (AFC), which is the number of antral follicles measured through transvaginal ultrasound. A systematic review revealed a negative correlation between BPA and AFC, as well as sex hormone-binding globulin (121). Animal experiments also showed that BPA exposure caused irregular estrous cyclicity and reduced the number of follicles at all levels, including AFC (122). Studies conducted in northern China and South Korea have further conveyed that BPA and bisphenol S (BPS) disrupt steroidogenesis, inhibit follicle growth, and heighten the risk of DOR (123, 124).

Alongside AFC, serum indicators like anti-müllerian hormone (AMH), follicle-stimulating hormone (FSH), and estradiol (E_2_) in serum are used to measure ovarian reserve. Despite certain studies reporting an inverse association between BPA and AFC and AMH, it is essential for multiracial clinical research to investigate the relationship between BPA and these markers of DOR (125).

##### PAEs

The EARTH Study investigated the effect of female exposure to PAEs on ovarian reserve, as measured by AFC. Mean AFC exhibited significant decreases with increasing concentrations of DEHP, with decreases of 24%, 19%, and 14% in the 2nd, 3rd, and 4th quartiles, respectively, compared to women in the 1st quartile of urine DEHP (126). Cross-sectional research conducted in China also revealed that PAEs exposure may lower ovarian reserve by downregulating the concentrations of corticosterone and cortisol in FF (127). Additionally, PAEs have been shown to enhance oxidative stress, leading to follicle death, faster depletion of ovarian reserve, and earlier reproductive senescence (128). *In vivo* and *in vitro* experiments found that DEHP treatment drives ovarian dysfunction by disrupting the normal expression of ovarian function-related genes, inhibiting the proliferation of granulosa cells, and reducing healthy follicles via the SLC39A5/NF-κB/NLRP3 axis (129).

Although less research has been conducted on the correlation between PAE exposure and the progression of DOR compared to BPA, increasing evidence from animal experiments indicates that certain PAEs can modulate endogenous steroid hormone signaling. Consequently, there is growing concern about accelerated follicle loss and reproductive aging in humans. Therefore, comprehensive analysis is necessary to examine the interplay between PAEs and markers of ovarian reserve.

##### Pesticides

Pesticides comprise semi-volatile persistent organic pollutants, categorized by their use as biocides, fungicides, bactericides, insecticides, and herbicides (130, 131). These pesticides have been widely used due to the increasing global population and the accompanying demand for larger food supplies. The widespread application of insecticides in the agricultural field as well as in household practices is one of the potential environmental risk factors affecting the function of women’s ovaries. Pyrethroids, a widely utilized class of insecticides, are typically evaluated through the detection of their metabolites in urine. Unconditional logistic regression analysis has revealed a significant correlation between the concentrations of 3-phenoxybenzoic acid (3-PBA), a well-known metabolite of frequently utilized pyrethrin insecticides, and an elevated risk of ovarian dysfunction in individuals with POI (132). Additionally, malathion, a broad-spectrum insecticide employed in agricultural settings and public health pest control, has been linked to oxidative stress, DNA damage, granulosa cell apoptosis, and autophagy, which contribute to reproductive toxicity (133).

Consequently, insecticides persist in the environment and enter organisms through various exposure routes, including ingestion, resulting in a profound reduction in follicle count. Moreover, these chemicals have the ability to disrupt the expression of genes related to ovarian function and modify epigenetic markers, thereby posing potential hazards to human reproductive health.

##### Other EDCs

Exposure to PFAS has been found to disrupt the initial stages of folliculogenesis and reduce ovarian reserve by interfering with ovarian enzyme activities involved in steroidogenesis or by inhibiting kisspeptin signaling in the hypothalamus (134). Greater exposure to PFOA has been observed in patients with DOR and poses a threat to embryo development by altering the metabolic composition of FF (135). Triclosan (TCS), a lipid-soluble phenolic compound with broad-spectrum antibacterial properties, can be exposed to the general population through dermal and mucosal contact with consumer products. A prospective cohort study including 109 women seeking fertility treatment found that urinary TCS concentrations were inversely associated with AFC, thus affecting ovarian reserve (136). A French case-control study detected 17 POPs in serum samples, with only p,p’-dichlorodiphenyldichloroethylene (DDE) being significantly associated with an increased risk of DOR (137). Another case-control study utilizing Bayesian kernel machine and logistic regressions, validated the combined and individual effects of EDCs on DOR, with a particular emphasis on the influence of benzophenone-4 (BP4) (138). In addition, PP biomarker concentrations were inversely associated with ovarian antral follicle count (139). Animal research has shown that parabens exposure can lead to changes in fertility, fecundity, and reproductive parameters, accelerating ovarian aging (140, 141). *In vitro* experiments have shown that PP exposure of mature ovarian follicles resulted in growth inhibition under culture conditions (142). A pilot study observed modest inverse associations between methylparaben and ovarian volume, such that ovarian volume was −4.28% smaller with every two-fold increase in methylparaben (143). However, an epidemiological study of humans reported methyl (MP), ethyl (EP), butyl (BP) and izobutyl paraben (iBuP) parabens were not associated any with parameters of ovarian reserve (139).

Exposure to EDCs has been observed to reduce the number of functional follicles in the ovary by activating specific signaling pathways, disrupting intercellular communication between oocytes and granulosa cells, and inducing oxidative stress. These effects ultimately result in DOR and have long-lasting detrimental impacts on reproductive function (123, 144). However, a study conducted on women attending a fertility clinic found no association between mixtures of phenols and PAE metabolites in urine and ovarian reserve (145). Due to the limited number of studies and inconsistent findings, the potential impact of these EDCs on adverse female reproductive outcomes remains uncertain.

#### EDCs accelerate reproductive aging causing premature ovarian insufficient (POI)

POI, defined as a loss of ovarian function, represents an intermediate stage between DOR and premature ovarian failure (POF). It is characterized by the premature depletion of follicles, leading to amenorrhea, subfertility, or infertility. POI is a complex process influenced by various factors, and the increasing incidence of this condition suggests that environmental factors may serve as primary causes alongside genetic factors.

##### BPA

The proposed role of EDCs in the development of DOR indicates that compounds such as BPA and other plasticizers pose a significant threat to reproductive health and contribute to reproductive aging. Studies have identified higher levels of BPA and mono-butyl phthalate (MBP) in the serum of women with POI, suggesting that these chemicals may act as contributing risk factors for POI (146). Exposure to BPA during fetal development disrupts the balanced activation of primordial follicles. This disruption leads to the depletion of the non-renewable pool of primordial follicles (PFs), while fetal exposure to BPA induces excessive endoplasmic reticulum stress in granulosa cells, accelerating primordial follicle activation and the subsequent development of POI (147). Numerous rodent studies have demonstrated that BPA can disrupt hormone cyclicity, likely through alterations in estrogen regulation, underscoring the significant impact of BPA on ovarian function. However, a study conducted in China measuring the concentration of BPA and serum levels of reproductive hormones found no significant association between BPA and FSH and AMH levels (148).

The inconsistencies in these findings can be attributed to the inherent limitations of retrospective case-control studies that rely on post-diagnostic assessment of exposure levels. It is crucial to note that the single sampling approach can still adequately reflect the average exposure of the population to BPA. Nevertheless, further evaluation through population-based studies conducted in occupational or environmental settings is needed to fully comprehend the associations between BPA exposure and its related health outcomes.

##### PAEs

PAEs are known to exert toxicity on the reproductive system and human development. The reserve of PFs, which represents an individual’s potential fertility, plays a major role in determining the female reproductive lifespan. Epidemiological studies and experimental research have indicated that both acute and chronic exposure to PAEs disrupt the estrous cycle, hastens the recruitment of PFs, and accelerates ovarian reproductive aging, ultimately leading to the development of POF (149). Clinical studies and preclinical findings demonstrate that PAEs and PAHs overactivate the calcium signaling pathway and the PI3K/Akt pathway, leading to the recruitment and depletion of PFs, ultimately precipitating the onset of POI (150). PAEs exposure also decrease the expression of AMH, and raise serum FSH levels, thereby increasing the risk of premature menopause and exacerbating the symptoms of POF (151). Additionally, a case-control study conducted in China indicated that higher urinary concentrations of PAEs may impair ovarian function and increase the odds of POF (152). Furthermore, findings from mouse studies indicate that prenatal exposure to environmentally relevant mixtures of PAEs accelerates biomarkers of reproductive aging, leading to decreased testosterone and inhibin B levels, elevated FSH and LH levels, and a decrease in the percentage of PFs, with these effects extending across multiple generations (153). In humans, dibutyl phthalate, which is associated with decreased hormone production and DOR, can reach the ovary and impact its function (154). Inflammatory factors, particularly tumor necrosis factor (TNF) production, have been proposed as significant contributors to the induction of apoptosis in the mammalian ovary by both natural and synthetic environmental estrogens such as di-2-ethylhexyl phthalate and BPA (155).

While the investigation of the effects of chemicals in PAEs on reproductive function is still limited, studies have demonstrated that PAEs hasten the activation and depletion of PFs, disrupt menstrual cycle, and influence reproductive aging. These findings suggest the involvement of epigenetic mechanisms underlying the impact of PAEs on these endpoints, but warrants further investigation is required to confirm this hypothesis.

##### Pesticides

Pesticides are considered to have an impact on neuroendocrine regulation of the gonadal axis and effects on ovaries at different levels. Case-control studies conducted on the female population in China have revealed a correlation between exposure to OCPs, particularly p,p’-DDT, and lower levels of AMH, with a dose-response relationship observed for the risk of POI (156). Female mice exposed to acceptable daily intake or chronic reference doses of cypermethrin (CYP) from gestational day 0.5 until 44 weeks old exhibit an ovarian phenotype resembling human POI, characterized by increased apoptosis, decreased cell proliferation, and downregulation of genes involved in steroidogenesis (157). Moreover, in a study involving sexually mature female rats, lambda-cyhalothrin (LCT), a type II pyrethroid pesticide, was administered at two different doses, resulting in a simultaneous reduction of gonadotropic hormone, estradiol, and progesterone levels, as well as degenerative changes in the ovaries (158). These findings indicate the reproductive effects of LCT.

It is noticeable that many of the pesticides are toxic to non-target organisms, including, aquatic organisms, birds, domestic animals, and particularly humans. Exposure to pesticides can potentially cause generalized oxidative stress, disrupt the secretion of gonadal hormones, and negatively impact oocyte quality, implantation rate, and pregnancy rate. Therefore, the government should strengthen the management of the agricultural environment, formulate a more detailed and perfect agricultural protection plan, and reduce the use of pesticides while solving agricultural pests.

##### Other EDCs

The compound 4-vinylcyclohexenediepoxide (VCD), which serves as a flame retardant, antioxidant, and plasticizer, has been found to selectively damage primordial and primary ovarian follicles, deplete the follicular reserve, and induce a mouse model of POI (159). Clinical case-control studies show that dioxin-like PCBs have been significantly associated with POI (156). 2,3,7,8-tetrachlorodibenzo-p-dioxin (TCDD), a by-product of organic synthesis and burning, can rapidly accumulate in human and animal tissues, leading to reduced fertility. Pregnant Sprague-Dawley rats exposed to TCDD exhibit diminished ovarian reserves, and the offspring of these rats show inhibited follicular development in adulthood (160). Systematic reviews of the literature and a knowledge synthesis also pointed out that TCDD alters estrous cyclicity and impairs follicular development (161). Zhang et al. suggested that PFOS exposure may suppress ovarian hormone production and impair follicular development, resulting in the loss of ovarian function and earlier menopause among POI patients (162). In addition, parabens accelerate ovarian dysfunction in a 4-vinylcyclohexene diepoxide-induced ovarian failure model (163).

Therefore, the increasing data from epidemiological studies and experimental animal models indicate that exposure to the above EDCs can exhaust and deplete follicular cells, resulting in an earlier age of menopause, POI, and even POF. As prospective studies on EDC exposure levels in humans are challenging, conducting large-scale animal or cell experiments in the future could potentially address this limitation and offer significant insights into the relationship between EDCs and ovarian function.

#### EDCs contribute to lower probabilities of implantation and clinical pregnancy

Infertility rates in humans have witnessed a significant increase worldwide since the turn of the 21st century. The impaired secretory function of the HPG axis and the morphology and function of the female reproductive system may ultimately lead to female infertility. Exposure to certain EDCs, either alone or in combination, can disrupt the endocrine system and compromise the integrity of human stem cells. As a result, difficulties in conceiving or carrying a pregnancy to term emerge, thereby establishing EDCs as crucial environmental risk factors for infertility (164, 165).

##### BPA

Both epidemiological and experimental evidence demonstrate that all bisphenols affects female infertility and subfertility (166, 167). This effect extends to BPA substitutes like BPS and bisphenol F (BPF), which exhibit parallel endocrine-disrupting effects. *In vitro* maturation experiments involving bovine cumulus-oocyte complexes (COCs) exposed to BPA or BPS for 24 hours present heightened spindle abnormalities in MII oocytes and chromosome misalignment across various concentrations, ultimately leading to infertility (168). BPS is present in culture media used in assisted reproductive technology (ART) and cell culture, which may contribute to a decrease in the success rate of ART (169). Interventions using human, mouse, and human granulosa cell line (KGN) models have demonstrated that BPA exposure can abnormally influence ovarian functions, leading to abnormal folliculogenesis through the activation of autophagy in granulosa cells via the AMPK/mTOR/ULK1 pathway (170). BPA-induced apoptosis, mediated by GPER-dependent activation of the ROS/Ca^2+^-ASK1-JNK pathway in KGN cells, has also been linked to the occurrence of female infertility (171). Data from the National Health and Nutrition Examination Surveys manifested that a combination of phenol and PAE metabolites is linked to infertility among reproductive-age women, particularly BPA and DEHP. And the probability of infertility increased dramatically as the quantiles of the total mixture concentration increased (172). However, some research has suggested that BPA exposure is not associated with increased embryo implantation failure, decreased fertilized oocytes, or decreased oocyte counts (173).

Collectively, most experiments indicated an impairment of female infertility with BPA, notably during perinatal. This impairment occurs through various mechanisms, including disruption of gonadotropin signaling, sex steroid hormone production, histone modifications, and miRNA expression. Additionally, BPA exposure during early life may have transgenerational effects. It would be more informative to evaluate BPA exposure prior to disease onset rather than at the time of diagnosis. Larger and more diverse epidemiological research is needed to refine the above finding.

##### PAEs

Significantly higher values of certain PAEs, particularly DEHP, suggest a possible involvement of these compounds as competing factors in reproductive issues, especially in women with idiopathic infertility (174). One study identified preliminary evidence suggesting an association between DEHP and infertility in women (175). Urinary PAE metabolite concentrations have been found to be negatively associated with AMH hormone concentrations in the FF of women undergoing fertility treatment. This association may have implications for antral follicle recruitment and fertility treatment outcomes (176). High DEHP exposure and its correlation with infertility have been confirmed in a study conducted in Jordan (177). Furthermore, DEHP metabolites in Sweden and Estonia have shown a significant inverse association with the ovarian sensitivity index, potentially leading to altered ovarian function and infertility in women (178). Studies have also indicated that paternal mixtures of urinary DEHP metabolite concentrations are related to higher rates of infertility treatment failure (179). Moreover, findings from studies using FF aspirated from individual follicles indicate that PAE-induced endocrine defects observed *in vitro* in follicular cells may also occur in humans at environmentally relevant exposure levels (180). Research conducted on Wistar rats has demonstrated that exposure to DEHP or diisononyl phthalate (DINP) can disrupt ovarian follicle recruitment and ultimately maturation, potentially resulting in severe consequences for female fertility (181).

Studies have identified preliminary evidence suggesting an association between DEHP and infertility in women may contribute to lower probabilities of implantation, clinical pregnancy, and live birth by changing estrous cyclicity, aggravating oxidative stress, and damaging oocyte quality. It is worth noting that the results of these studies should be interpreted with caution due to the limitations of different research subjects, methods, and quantities.

##### Heavy metals

Environmental degradation can increase the likelihood of human exposure to heavy metals (HM) from natural sources and human activities. Such exposure can lead to various health consequences, including reproductive problems. A cross-sectional analysis from 2013 to 2018 NHANES data revealed urinary arsenic (As) and cadmium (Cd) were associated with female infertility, while blood/urine lead (Pb) was found to be related to infertility in overweight/obese women and those in advanced age (182). Cd has been shown to induce apoptosis of the human granulosa cell line KGN through mitochondrial dysfunction-mediated pathways and may cause female reproductive toxicity (183). Pb exposure affects ovary development, folliculogenesis, and steroidogenesis in rats through the activation of the IRE1α-JNK signaling pathway, posing a hazard to reproductive health (184). The microenvironment provided by the follicular fluid (FF) affects the quality of oocytes. An excess of iron and calcium in the FF reduces the rate of good quality embryos, while an excess of potassium impairs the blastocyst rate (185). Increased use and improper disposal of hexavalent chromium (Cr), an environmental contaminant, can disrupt oocyte development in rats by elevating oxidative stress, causing DNA double-strand breaks, microtubule disruption, and aberrant chromosome segregation. These effects may result in embryo lethality, infertility, or birth defects (186). In addition, mercury impairs the function of human primary endometrial stromal cells, impacting human fertility and posing a hazard to the outcomes of IVF procedures (187).

Exposure to heavy metals is known to have detrimental effects on oocyte maturation, ovulation, and fertilization. This occurs through the induction of oxidative stress and damage to biological molecules, ultimately impacting female reproductive capacity and assisted reproduction treatments. Although the precise causal link remains incompletely understood, these findings have significant implications for the management of patients with subfertility. To mitigate potential harm to the reproductive system, it is crucial to minimize exposure to metal pollutants in daily life and carefully consider the intake of specific trace elements.

##### Other EDCs

Apart from the aforementioned BPA, PAEs, and heavy metals, numerous other EDCs have detrimental effects on reproductive health. Glyphosate, the primary ingredient in glyphosate-based herbicides, has been shown to disrupt female reproduction in various animal models including fish, chicks, rats, mice, and ewe lambs. Its effects can include prolonged time-to-conceive, spontaneous abortion, stillbirths, and developmental defects (188, 189). These impacts are believed to be mediated by alterations in estrogen receptors and molecules involved in estrogenic pathways. Chlorpyrifos (CPF), a very effective, low-cost, and easily accessible acaricide insecticide, either as a consequence of hormonal changes or by direct local action, induces proliferative changes in the uterus of the rat (190). This result may suggest that CPF chronic exposure could affect reproduction or act as a risk factor in the development of uterine proliferative pathologies. Endosulfan, one of the major cyclodiene pesticides, is also shown to cause ovarian regression in females, alterations in hormone synthesis, follicular maturation, ovulation process, and ovarian cycle, which leads to an increase in infertility (189, 191). A population-level study revealed a heightened correlation between pesticide metabolite concentrations in urine and the risk of infertility among individuals with a high body mass index (192). Additionally, a 12-month prospective follow-up study involving 1,182 couples attempting to achieve pregnancy discovered an elevated risk of menstrual abnormalities and low fertility associated with high levels of TCS, a common ingredient in personal care and household products (193). The concentration of polypropylene in the urine of infertile women, determined through verified gas chromatography ion volley mass spectrometry, has been found to be related to ovarian reserve parameters (139). This suggests that polypropylene may have the potential to reduce fertility. Analysis of POPs in the blood and FF collected from infertile women undergoing assisted reproductive technology treatments in Sweden has revealed an association between HCB levels and clinical pregnancy and live birth outcomes (194). Subsequent studies have found that higher concentrations of PCBs and pesticides in FF are associated with thinner endometrial thickness, reduced numbers of retrieved oocytes, and decreased fertilization rates in intracytoplasmic sperm injection procedures (195). Self-reported lifestyle factors and EDCs concentrations in FF have shown that the presence of PFAS increases ovarian sensitivity to hormone stimulation while reducing embryo quality and fertility (196, 197), especially preconception exposure (198). *In vitro* models examining the effects of DES on the human ovarian cortex provide compelling evidence that it decreases the density of unilaminar follicles and negatively impacts fertility (199). Additionally, studies using environmentally relevant doses of TBT to disrupt mammalian ovarian function *in vitro* and *in vivo* have demonstrated impaired ovarian function and fertility due, in part, to abnormal sensitivity to steroid feedback mechanisms and detrimental effects on the ovaries (200, 201). Ethyl paraben and mixtures of benzophenones, TCS, and BPA were associated with infertility among women in the United States (202). Within a large chemical mixture, inverse associations levels of DEHP metabolites and methylparaben, and possibly PFOA, with ovarian sensitivity index are observed in Sweden and Estonia women, suggesting that these chemicals may contribute to altered ovarian function and infertility (178). Interestingly, the study conducted in Asians announced that the joint effect of paraben mixture on couple fecundity was non-significant (203).

There is convincing evidence that EDCs can potentially harm human health and reproductive potential. The above-mentioned studies have demonstrated that oocytes in the FF can be directly exposed to these chemicals, resulting in endocrine-mediated effects that can lead to ovulation failure. Furthermore, certain EDCs have been shown to cause endometrial dysfunction and implantation failure, potentially through the involvement of oxidative stress, inflammatory and apoptotic mediators. These effects ultimately reduce pregnancy rates and pose a threat to female reproductive function. It is imperative to advocate for sensible lifestyle modifications in both females and males aiming to conceive, in order to minimize EDC exposure. To sum up, the results of these studies should be validated in-depth, and the differences in the body’s response and the interaction between the environment and genes need to be clarified through subsequent prospective population research.

#### EDCs interfere with some endocrine and intracrine targets relevant to breast cancer (BC)

The mammary gland serves as the targeted regulatory organ of the hypothalamic-pituitary-ovarian axis. Imbalances in this axis can lead to hormonal secretion disorders, which in turn affect the breast by causing mutations in cell and tissue proliferation and division. These alterations also impact the expression of oncogenes and tumor suppressor genes, ultimately contributing to the pathogenesis of BC. BC, a hormone-dependent specific tumor of the reproductive system, accounts for approximately 30% of female cancers and represents one of the leading causes of death in women (204, 205). The secretion of estrogen and progesterone from the ovaries stimulates the proliferation of myoepithelial cells within ducts, with estrogen being the primary sex hormone responsible for mammary gland development during critical life stages (206). Risk factors for BC can be categorized into familial, hereditary, and environmental exposure. The estrogenic properties of EDCs may be potentially linked to the increasing rates of BC. Demographic research has confirmed the relationship between the incidence of BC and EDCs, such as BPA, PAEs, pesticides, parabens, polybrominated diphenyl ethers (PBDEs), and other environmental estrogens compounds.

##### BPA

Research indicates that the mean concentration of BPA is higher in cancerous patients compared to non-cancerous individuals, potentially increasing the risk of BC incidence (207). Following in-depth research reported that BPA may induce metastatic aggression in low metastatic human BC cells through PGC-1α mediated mitochondrial biogenesis and epithelial-mesenchymal plasticity (208). Notably, co-exposure to DEHP and BPA increased the incidence and reduced the latency of mammary tumors, which seemed to enhance the susceptibility of carcinogen-induced tumors (209). Despite DEHP exhibiting no apparent estrogenic activity, it can still disrupt ER activity to increase serum estradiol levels. Nevertheless, a recent meta-analysis, comprising 9 case–control studies involving 7820 BC cases, found no associations between BPA and BC (210).

Large-scale epidemiological studies are essential to determine the causal relationship between BPA and BC. The ER is a key transcriptional factor that drives the oncogenesis and growth of hormonally sensitive breast cells in collaboration with other growth factors. At least, BPA exposure could be a risk factor for ductal hyperplasia, possibly through an estrogen-dependent mechanism.

##### PAEs

Studies have indicated a positive association between PAEs and BC risk (211). Differential increases in serum PAE concentrations have been observed in women with BC compared to those without the disease in Mexico and Toluca City, thereby supporting the hypothesis of a positive association between PAE exposure and BC incidence (212). It has been suggested that PAEs could exert multiple parallel interactions with ERα signaling, emphasizing adverse health outcomes in BC cell proliferation (213). Furthermore, evidence indicates that the activation of the aryl hydrocarbon receptor (AhR) can promote cancer cell metastasis. Both MEHP and TCDD can induce migration and invasion in MCF7 human BC cells, with this promotion being partly AhR dependent (214). What’s interesting is that the co-exposure can produce antagonistic effect. Moreover, other authors found that PAEs metabolites could upregulate the expression of disintegrin and metalloproteinase domain 33 (ADAM33), which plays a significant role in reducing BC risk (215).

As a commonly used plasticizer, PAEs are widely utilized in industrial, medical, and life-related fields, and are considered to be ubiquitous environmental hormones. The complex underlying mechanisms of BC development, the wide variety of PAEs, and ethnic differences in study populations may have contributed to the contrary findings.

##### PBDEs

Polybrominated diphenyl ethers (PBDEs) are synthetic halogenated compounds that have been widely distributed as environmental contaminants due to their extensive use as additive flame retardants in various household products, such as furniture and hard plastic coatings in appliances. Owing to their endocrine-disrupting effects, PBDEs have garnered significant interest in their potential connection with BC. Certain PBDE congeners have been shown to stimulate the proliferation of human BC cells (216). Recent studies involving Chinese women have revealed a positive correlation between BC growth and exposure to BDE-47, one of the PBDE congeners that has received intense focus due to its widespread existence in the environment and adverse health effects (217, 218). However, evidence from case-control studies in France and California has shown no significant associations between PBDEs and BC (219, 220). Additionally, significant differences in PBDE levels were not observed when examining malignant and benign tumor tissues (221).

Due to their structural similarities to PCBs, PBDEs are also considered suspected human chemical carcinogens. Although the mechanism by which PBDEs cause cancer is not well understood, efforts should be made to minimize daily exposure, as they enter the food chain and accumulate in higher predators that are eventually consumed by humans.

##### Pesticides

When considering the links between breast cancer (BC) and environmental factors in humans, the most compelling data, apart from diet, arises from pesticide exposure in farming, which has been associated with an increased incidence of BC. The mammary glands of both male and female rats and mice are susceptible to non-organochlorine (such as vinclozolin, atrazine, glyphosate, chlorpyrifos) and organochlorine (including endosulfan, methoxychlor, hexachlorobenzene) pesticides (222, 223). Experimental models have demonstrated various effects of these compounds, including alterations in mammary development, impaired cell proliferation and steroid receptor expression and signaling, heightened malignant cellular transformation and tumor development, and increased angiogenesis.

So far, most studies have focused on individual pesticides or the direct modulating effects of compounds on sex hormone receptors. Few prospective studies have investigated OCPs in relation to overall or BC-specific mortality following the diagnosis of BC. Therefore, evidence from *in vivo* and epidemiological studies needs to be further expanded.

##### Parabens

Epidemiological evidence also suggests a connection between paraben exposure and the development of BC. For instance, one study indicated that the highest quintiles of urinary total parabens in women were linked to an increased risk of BC (224). Additionally, parabens have been detected in breast tissue, including breast tumor tissue (225, 226). Specifically, parabens have been observed to enhance the proliferation of BC cell lines at biologically relevant levels, mainly in an ER-dependent manner (227). Mechanistic research has also revealed that exposure to PP during pregnancy and lactation increases epithelial cell proliferation, decreases collagen thickness, and alters the immune cell profile in the mammary gland (228). However, conflicting conclusions have been reported in some studies. For instance, a case-control study involving a multiethnic cohort showed an inverse relationship between total paraben exposure and BC risk in postmenopausal women (229). It is worth noting that one major difference between these studies is the collection of post-diagnostic urinary samples in one case and pre-diagnostic urinary samples in the other, leading to potential difficulties in comparing the results. Furthermore, contradictory findings have emerged from studies examining the association of personal care product use (a potential source of parabens) and BC risk, with a weak inverse association found in the Norwegian Women and Cancer Cohort (230) and a 10% to 15% higher risk of BC in moderate-to-frequent users of beauty products compared to less frequent users in the Sister Study (231).

Research increasingly suggests that exposure to low doses of parabens during sensitive developmental periods can lead to changes in mammary gland morphology and gene expression. EDCs encountered during critical periods of breast cell proliferation and differentiation, such as puberty and pregnancy, have the potential to affect mammary gland development, but further elucidation of this relationship is necessary (232). The main concerns regarding parabens use in consumer products are their potential mimicry of endogenous hormones (226), possible cross-talks with other signal transduction pathways that are pivotal in the development of BC (233), and modulation of key enzymes involved in local estrogen metabolism.

##### Other EDCs

The World Health Organization (WHO) and the Endocrine Society have both highlighted the carcinogenic role of dioxin. A prospective cohort study in the USA suggested a positive relationship between municipal solid waste incinerator (MSWI)-produced dioxin exposure and invasive BC, with a higher risk for women living within 5 km of MSWIs (234). However, a case–control study nested within the French E3N prospective cohort did not show an increased risk of breast cancer from higher airborne dioxin exposure, possibly due to the small population size (235). Given that dioxin is classified as a known human carcinogen by the International Agency for Research on Cancer (IARC) and its pathogenic mechanism is plausible, it is reasonable to propose a possible role in breast cancer development. Additionally, exposure to PFOA promotes proliferation, migration, and invasion potential in human breast epithelial cells (236). Positive associations between breast cancer risk and perfluoroalkylated substances, including PFOA, have been observed in French women (220). However, a nested case-control study among California teachers showed no correlation between serum PFAS levels measured after diagnosis with breast cancer risk (237). Specifically, circulating PFOS concentration was associated with estrogen receptor–positive tumors, while low concentrations of PFOS and PFOA were associated with estrogen receptor–negative tumors (220). In other words, women younger than 50 years old had a higher risk, and this risk increased when tumors were estrogen receptor–positive. A follow-up study suggested that the inhibition of mammary gland development by PFOA might elevate the risk of developing mammary tumors through the activation of signaling pathways associated with tumorigenesis (238).

In addition, the association between DDT or its metabolites in lipid, serum, or plasma and BC incidences is noteworthy. Higher DDE and DDT levels were linked to worse overall survival in the Carolina Breast Cancer Study (239). Studies have shown that intrauterine and infant DDT exposure increases the risk of premenopausal BC, while DDT exposure after infancy elevates BC risk in the early postmenopausal years (240). This emphasizes that time of first exposure and the levels of exposure at younger ages are critical for accurate assessment of the BC risk. On the contrary, a cross-sectional study investigating the potential impact of organochlorine chemicals on mammographic breast density found no correlation between higher DDE levels in women and BC risk (241). Furthermore, a hospital-based case-control study in Chinese women found a positive association between certain PCB exposures and elevated incidence of BC (242). Moreover, a meta-analysis highlighted the increased risk of BC associated with five specific polychlorinated biphenyls (PCB 99, PCB 105, PCB 118, PCB 138, and PCB 183) (223).

Regarding Cd, a recent meta-analysis of cohort studies discovered only a marginal positive relationship between dietary cadmium intake and BC, with no clear mechanism (243, 244). *In vitro* and animal model studies have demonstrated that this heavy metal possesses estrogen-like properties, can increase migration and epithelial–mesenchymal transition of BC cells, and can promote the production of active oxygen species (245, 246). It is important to note that developing breast tissue is susceptible to select EDCs during childhood and adolescence. Polycyclic aromatic hydrocarbons (PAHs), a class of EDCs, are believed to potentially delay the onset of breast development in girls (247). PAHs are byproducts of incomplete combustion of coal, oil, natural gas, wood, and paper. Notably, it is particularly noteworthy that more than 150 types of PAH have been detected in the smoke emitted by smokers so far. PAH-enriched EDC mixtures have been shown to enhance aryl hydrocarbon receptor and antiapoptotic signaling, promoting a proliferative phenotype in BC cells (248).

Given that most studies on the relationship between EDCs and BC risk are case-control studies, it is inevitable that there may be potential biases in exposure estimates during sample collection. Prospective studies focusing on the association between duration, dose, and age of EDCs exposure, and BC risk should be considered. Additionally, the influence of androgen levels is also significant in BC development, and it is crucial to explore the effect of EDCs on testosterone and BC risk. For now, caution should be taken when individuals, including BC patients or individuals with a high risk of BC, make decisions on common products containing those EDCs.

#### EDCs facilitate the development of estrogen-dependent endometrial cancer (EC)

EC is the sixth most commonly diagnosed cancer in women. The most prevalent symptom is abnormal uterine bleeding, while vaginal discharge and pyometra can also occur. Prolonged exposure of the endometrium to estrogen in the context of a dysfunctional HPG axis and deficient progesterone conversion can facilitate the development of endometrial hyperplasia and potentially EC. The majority of ECs are estrogen-dependent type I cancers, and excessive exposure to EDCs heighten the associated risk.

Chronic exposure to BPA has been shown to impair steroid hormone signaling in the uterus of mice, potentially contributing to the development of uterine hyperplasia and cancer (249). Research on the risks of EDCs to EC has been extensive. Apart from the receptor-mediated mechanism, BPA may exert its carcinogenic role through epigenetic mutations, thereby altering developmental pathways and cellular processes (250). A low-dose BPA-gene signature has demonstrated predictive value for survival outcomes in EC patients (251). Analysis using inductively coupled plasma optical emission spectrometry has explored risk factors for EC and blood cadmium (Cd) levels, indicating that intake or environmental exposure to Cd may result in estrogen-dependent diseases, including EC (252, 253). Moreover, high dietary Cd intake might be associated with poorer outcomes in women with EC (254). With growing evidence suggesting a relationship between altered serum trace metal concentration and EC, further research on a larger group of patients is required to validate and evaluate our findings. Especially regarding serum copper and zinc (Zn) concentrations, which have shown a potential link to altered trace metal levels and EC (255, 256). A nested case-control study conducted within the Multiethnic Cohort revealed a positive association between the urinary excretion of monoisobutyl phthalate (MiBP) and MnBP and the risk of EC (257). In addition, the occurrence of EC was found to be associated with the use of alkylphenol ethoxylates (APEOs), nonionic surfactants widely utilized as emulsifiers (258).

Mounting evidence suggests a link between EDCs and the incidence and development of hormone-related tumors, with EC serving as a representative example. Suspected EDCs, including BPA, MiBP, MnBP, and APEOs, are thought to induce genotoxicity via multiple pathways, thereby increasing the rate of EC development. Furthermore, excessive accumulation of non-essential heavy metals like copper in the body has been linked to the occurrence of EC. Due to the burden of gynecological cancer on female reproductive health, additional studies are urgently needed to identify risk factors. The prevention and treatment of EC should be based on reducing exposure to the above EDCs.

#### EDCs induce cell aberrant proliferation and metastasis in ovarian cancer (OC)

The available data have shown that OC is associated with a number of estrogen-regulated pathways, similar to CC, which are also partially regulated by HPG axis function. The concealment of early symptoms of OC, coupled with the lack of accurate non-invasive screening methods, contributes to more than 70% of patients have progressed to advanced stages when diagnosed, resulting in OC accounting for over two-thirds of gynecological cancer-related deaths (259). Multiple factors, including genetic, endocrinology and environmental, may contribute to the process of OC formation, of which EDCs are an established risk factor.

Gene Ontology analysis revealed that exposure to 100 nM BPA resulted in the dysregulation of numerous genes, specifically 159 genes associated with OC (260). In silico analyses utilizing gene expression data from The Cancer Genome Atlas (TCGA) and the Genotype-Tissue Expression (GTEx) databases demonstrated that 14 out of 94 genes were exclusively dysregulated in the presence of BPA, while the remaining 80 genes already exhibited dysregulated expression patterns as a consequence of the disease itself (261). Although EDCs do not typically modify DNA sequences, each category of EDC has its specific mechanism of action, which can differ from others. Heavy metal Cd increases the expression of P-glycoprotein in the ovarian cell line SKOV-3, which may negatively affect anticancer treatment (262). Benzophenone-1 (BP-1), an endocrine-disrupting compound found in personal care products, exhibited estrogen-like effects. Molecular dynamics simulations, in combination with yeast-based assays, demonstrated the persistent binding of BP-1 to the ligand binding domain of ERα, leading to the abnormal proliferation and metastasis of OC cells (263).

These studies underscore the potential role of EDCs with sex-hormone activity in the etiology of OC. Despite being relatively uncommon, OC is a highly lethal disease. Exposure to EDCs such as BPA, Cd, and BP-1, commonly found in plasticizers, cosmetics, and other products, may contribute to the progression of OC through epimutations. The diverse range of changes observed with different compounds suggests the involvement of multiple mechanisms in promoting female reproductive pathology. These findings provide insights for implementing preventive measures aimed at reducing exposure to the mentioned EDCs, subsequently affecting disease progression.

#### EDCs regulate the cell cycle in cervical cancer (CC)

CC, along with EC and OC, has a potentially impaired HPG axis function, leading to abnormal hormone secretion pulse rhythms, and is the most commonly diagnosed cancer among women and a leading cause of cancer-related deaths. While human papillomavirus (HPV) is a necessary but insufficient cause of CC, other significant cofactors, including certain EDCs, also play a prominent role. High levels of BPA and PAEs exposure have been observed in women with CC, with the metabolite MEHP, a byproduct of DEHP, exhibiting the highest concentration (264). Additional research is needed to investigate the potential synergistic proliferation of CC resulting from the interplay between EDCs and HPV, given the correlation observed between urine samples from CC women and HPV infection.

The regulation of cancer gene expression involves a cooperative interplay between epigenetics and genetics. EDCs not only have the ability to regulate hormone levels in the body through HPG but also can affect the biological processes of OC through various pathways such as epimutations. There are still significant knowledge gaps in understanding the correlation between OC and EDCs due to the multifactorial nature of cancer development.

Aside from BC, EC, OC, and CC, tumors can also emerge in other gynecological sites such as the vagina and fallopian tubes. EDCs, particularly BPA, present various threats to the occurrence and progression of gynecological cancers. However, determining the carcinogenic effects of EDCs is challenging due to the difficulty in evaluating lifelong exposure. While adults typically require higher concentrations of EDCs to experience toxic effects, developmental stages can be affected by low doses, and cumulative exposure to the environment can lead to the manifestation of diseases later in life. The exact amounts of EDCs causing cancer are still unclear, but it is hypothesized that during the embryonic period, puberty, menopause, and when the ovaries are in a degenerating state, they may be more vulnerable to the effects of exogenous chemicals in the form of EDCs. In other words, EDCs can have an impact even at low doses and can induce unpredictable interactions, thus establishing their recognition as potential carcinogens. Conducting additional studies using ex vivo and *in vivo* models may help bridge knowledge gaps regarding the effects of BPA, other EDCs, or their mixtures on carcinogenesis.

### Research on EDCs in the male reproductive system

**Figure 3:**
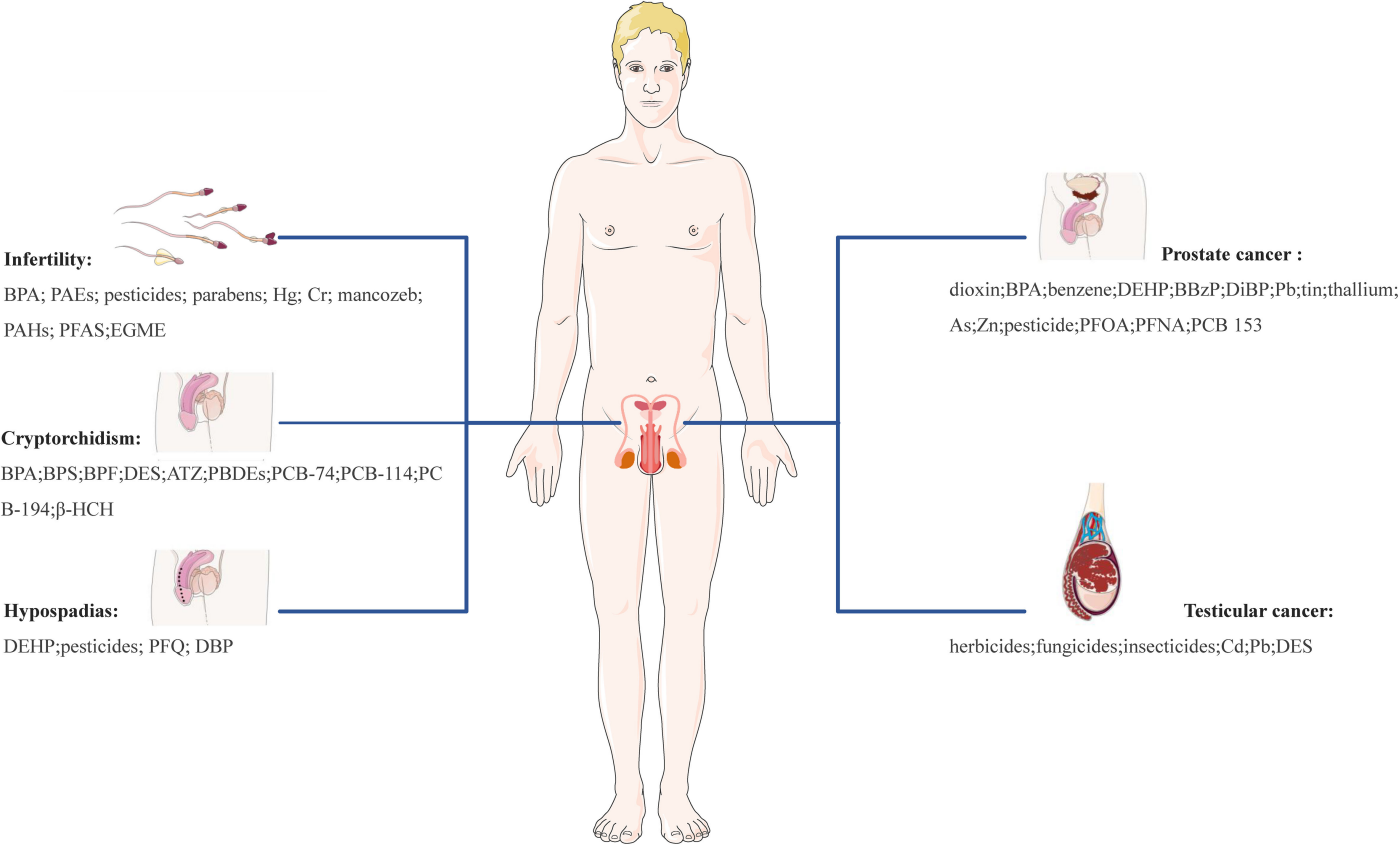
Effect of EDCs on male reproductive health. EGME, ethylene glycol monomethyl ether; ATZ, atrazine; PBDEs, polybrominated diphenyl ethers; β-HCH, β-hexachlorocyclohexane; PFQ, pyrifluquinazon; DBP, dibutyl phthalate; PFNA, perfluorononanoic acid.

EDCs negatively affect the female reproductive system through various mechanisms including, but not limited to, the regulation of the hypothalamic-pituitary-ovarian axis. Similarly, the male reproductive function is primarily regulated by the hypothalamic-pituitary-testicular axis. EDCs affect hypothalamus that stimulates pituitary gland to secrete Gn, which regulate spermatogenesis through Leydig cell androgen secretion and Sertoli cell activation. EDCs can also negatively impact the function of the male reproductive organs, particularly the testis, which is crucial for spermatogenesis and male hormone secretion. EDCs can affect spermatogenesis, compromise sperm quality, and contribute to the onset of Testicular Dysgenesis Syndrome (TDS). TDS encompasses conditions like cryptorchidism, hypospadias, and testicular changes that can increase the risk of developing cancer. Importantly, more and more evidence showed that EDCs, such as BPA, DES, PCB, and pesticides, can also be involved in the development of TDS and other male reproductive system-related disorders, even cancer, posing a substantial threat to male reproductive health. A comprehensive overview of common EDCs related to male reproductive system diseases is shown in **Figure 3**.

#### EDCs damage semen quality and lead to male infertility

Infertility affects approximately 15% of the global population, with male factors responsible for 40–50% of these cases (265). The increasing incidence of male infertility can be attributed to various factors, including hormonal imbalances, anatomical causes, sexually transmitted diseases, genetic, environmental and lifestyle factors (266, 267). While the mechanisms leading to male infertility are not fully understood, changes in hormonal balance, particularly during sperm development, may impact sperm quality (268). Since the hypothalamic-pituitary-testicular tightly controls male reproductive system, EDCs acting as anti-androgens or mimicking estrogens may interfere with these mechanisms and have a profound impact on spermatogenesis. Experimental evidence suggests that increased exposure to EDCs may contribute to the decline in sperm counts, as well as impaired sperm motility and morphology (8, 269–271). Furthermore, the analysis of semen parameters, such as sperm count, motility, and morphology, provides valuable insights into testicular function and serves as a tool in fertility evaluation.

##### BPA

BPA can have a negative impact on sperm quality and directly affect seminal plasma, thereby influencing the testicular environment (272). Studies using animal models and *in vitro* methods have revealed that exposure to BPA is linked to decreased sperm count, impaired sperm motility and velocity, reduced epididymis weight, impaired insulin signaling and glucose regulation, and lower levels of Gn, testicular steroids, and testosterone in the serum (273–275). A study involving 74 and 83 male workers from factories with and without occupational exposure found that increased BPA exposure was associated with higher hydroxymethylation levels, suggesting potential adverse effects on male fertility (276). Furthermore, studies on reproductive-aged Chinese men have shown an association between BPA and altered serum hormone levels, such as low testosterone levels, ultimately leading to reduced male fertility (277, 278). Low testosterone levels are a major cause of male infertility, as this hormone is crucial for most processes throughout the entire male reproductive tract. However, a study involving 146 couples undergoing *in vitro* fertilization found that BPA concentrations did not significantly impact sperm concentration or motility parameters (279). Similarly, no statistically significant association was found between BPA concentration in the urine and parameters of seminal fluid (280, 281).

The correlations between BPA exposure and fertility in different populations of males have been investigated, but the results remain contradictory and inconclusive (282–284). The inconsistent findings are possibly due to the variations in BPA exposure levels. While male infertility cannot solely be attributed to BPA exposure, higher concentrations of BPA may contribute to infertility (285). Additionally, in epoxy resin workers occupationally exposed to BPA, alterations of sperm LINE-1 hydroxymethylation were observed (286). Furthermore, a Chinese cohort study found that hypermethylation of LINE-1 was significantly associated with tobacco smoking in men at risk of infertility (287). Therefore, daily exposure to BPA through lifestyle factors such as smoking can regulate male hormones through the HPG axis or damage sperm quality through epigenetic modifications, potentially leading to male infertility. In the future, multiple sample studies should be conducted under standardized BPA exposure levels, and men should be encouraged to develop healthy lifestyles, such as quitting smoking, to avoid unnecessary BPA exposure as much as possible.

##### PAEs

The serum levels of PAE metabolites have been identified as a reliable predictor for male infertility (288. Numerous studies have consistently reported a positive association between urinary levels of PAEs and at least one semen parameter indicative of low semen quality, with some studies also indicating sperm DNA damage (289). Paternal mixtures of urinary concentrations of DEHP have been relevant to higher infertility treatment failure (179), providing compelling evidence of the link between PAE metabolite levels and decreased sperm quality. Additionally, environmentally relevant concentrations of specific PAEs in seminal plasma have been linked with reduced semen volume, sperm motility, viability, and morphological alterations in sperm heads, resulting in semen volume and sperm viability falling below reference values (290).

Moreover, mechanistic research suggests that sperm protamine serves as a molecular biomarker during spermatozoa apoptotic processes, potentially mediating the association between PAE exposure and spermatozoa apoptosis (291). Furthermore, lipophilic phthalic acid esters have been found to impair the human sperm acrosomal reaction likely through the inhibition of the phospholipase A_2_-signaling pathway (292). MEHP has been shown to induce telomere structure and function disorder, mediating cell cycle dysregulation and apoptosis through c-Myc and its upstream transcription factors in a mouse spermatogonia-derived cell line (293). In addition, chronic dietary administration of lower levels of diethyl phthalate has been observed to induce murine testicular germ cell inflammation and sperm pathologies, highlighting the undesirable male reproductive outcomes following chronic DEP exposure (294). In conclusion, the levels of PAEs and their metabolites in serum, urine, and seminal plasma are closely related to sperm parameters. Detecting PAEs in infertile men may clarify the mechanisms of PAEs causing sperm toxicity and provide credible evidence that PAEs could pose a serious threat to male infertility by inducing sperm apoptosis in the future.

##### Pesticides

Evidence from one research suggests that serum concentrations of multiple OCPs may be correlated with altered serum testosterone levels, indicating potential adverse effects of pesticide exposure on male reproductive health (295). Numerous studies consistently demonstrate associations between pesticide exposure and compromised sperm parameters, particularly sperm motility and sperm DNA integrity (296). Notably, ancestral exposure to p,p’-DDE has been linked to impaired testis histology, decreased the sperm fertility in the F1 generation, and transgenerational sperm DNA hypomethylation of paternally imprinted genes (297). DDE, a metabolite of organochlorine insecticide DDT, has been shown to have anti-androgenic effects. In a cross-sectional study of Chinese men, higher levels of serum γ-hexachlorobenzene (γ-HCH), a seed mixing and sterilization agent, were correlated with decreased sperm motility, and this association remained statistically significant even after adjusting for multiple testing (298). Further research confirms that HCH impairs human sperm motility by affecting lysine glutarylation and mitochondrial functions (299). Moreover, a human investigation reported that urine concentrations of pyrethroid metabolites were linked to elevated rates of sperm aneuploidy (300, 301). Exposure to organophosphate pesticides has also been found to decrease sperm count, concentration, total and progressive motility, and normal sperm morphology, potentially via a testosterone-independent mechanism (302).

Extensive *in vitro* and *in vivo* studies have been conducted to investigate the effects and potential mechanisms of pesticides on the male reproductive system, uncovering a spectrum of negative impacts that may ultimately culminate in infertility (303). Widely used globally, carbamates have been found to disrupt various signaling pathways, such as those mediated by acetylcholine or kisspeptin, compromising steroidogenesis and spermatogenesis (304). The benzoylurea pesticide Lufenuron has been associated with significant histological and histochemical damage in mammals, resulting in impaired testicular function and subsequent infertility (305). Additionally, dimethoate (DM), a prominent organophosphate insecticide used for pest control in vegetables, fruits, and agricultural crops, has been found to have detrimental effects on sperm morphology, fertility, and embryos, posing potential health risks to humans and non-target organisms due to its persistence in soils and crops (306). Furthermore, amitraz (AMZ), a formamidine pesticide widely used as an insecticide and acaricide, has been shown to induce alterations in bovine sperm, potentially affecting male fertility at concentrations found in the environment (307). Similarly, exposure to other pesticides such as diazinon (DZN) (308), pyridaben (PDB) (309), bifenthrin (BF) (310), flurochloridone (FLC) (311), fipronil (FPN) (312), etoxazole (ETX) (313), imidacloprid (IMI) (314, 315), atrazine (ATR) (316, 317), cypermethrin (CYP) (314, 318), chlorpyrifos (CPF) (314, 319–322), chlorpyrifos (323, 324), simazine (325), endosulfan (191), and deltamethrin (318) could lead to a significant decrease in sperm count, motility, normality, vitality, and an increase in deformity rates. Moreover, a significant decrease in daily sperm production, epididymal sperm count, motile, viable, and hypo-osmotic swelling-tail swelled sperm was observed in mancozeb-treated rats (326). Mancozeb, a contact fungicide and a polymeric complex of manganese and zinc salts of ethylene-bis-dithiocarbamate, is linked to histological alterations in the testis and a decrease in testosterone biosynthesis. Additionally, piperonyl butoxide (PBO), a semi-synthetic methylenedioxyphenyl compound, is commonly used as a synergist to enhance the insecticidal effect of pesticides for agricultural and household use. Studies have indicated that PBO considerably reduces sperm motility, kinematics, and acrosome-reacted and capacitated spermatozoa (327). The use of the pesticides and their synergists may directly or indirectly cause disorders in male fertility. Furthermore, exposure to these pesticides can trigger inflammation, mitochondrial defects, DNA damage, and epigenetic gene methylation, resulting in toxic and pathological effects on cells related to the reproductive system and causing lasting and serious consequences for reproductive organs and male reproductive health. Therefore, greater efforts are warranted to protect ourselves and our environment from the detrimental effects of these pesticides.

The understanding of the potential molecular mechanism of pesticide-induced toxicity on sperm provides a theoretical basis for the future treatment of male infertility. The findings unequivocally demonstrate that pesticides can have detrimental effects on semen quality, which in turn impairs the normal functioning of male reproductive function. Moreover, a study examining the effects of environmental pyrethroids on sperm morphology in men with oligozoospermia further consolidates this point (328, 329). Additionally, epidemiological evidence supports the correlation between pesticides and male fertility, particularly in relation to semen quality, DNA fragmentation, and chromosome aneuploidy among workers and the exposed population (330). It is hypothesized that pesticides may impact fertility by directly affecting spermatogenesis through reduced antioxidant capacity, impairment of testicular production of testosterone, systemic alterations in testosterone metabolism, mitochondrial dysfunction, or by influencing the HPG axis to steroidogenesis and inducing changes in epigenetic modifications.

##### Other EDCs

In various *in vitro* and *in vivo* studies, parabens have been associated with weak estrogenic and anti-androgenic actions (331). One study demonstrated that paraben use during pregnancy results in significant changes in the mitochondrial bioenergetics and antioxidant capacity of testicular germ cells, as well as the antioxidant capacity of several F1 generation organs, contributing to the understanding of male infertility in future generations (332). Parabens, especially butyl, can affect the enzymatic antioxidant system in rat testes, leading to a decrease in cellular antioxidant capacity, which may be related to male infertility (333). However, a couple-based prospective cohort study showed paraben exposure in male partners was not associated with fecundity (203).

Heavy metals cause toxicity by impacting the HPG axis, testicular function, spermatogenesis and sperm processing. Higher levels of Hg are associated with decreased semen quality and higher levels of DNA damage in men and lead to lower fertility and higher spontaneous abortion rates (334). Cr III, classified as an endocrine-disrupting chemical, and its compound Cr III picolinate (CrPic3), impact male fertility by altering reactive oxygen species, testosterone levels, and sperm parameters, such as sperm motility and abnormal sperm count (335).

A meta-analysis of 272 occupationally exposed men and 247 unexposed men found a significant decrease in sperm motility in participants occupationally exposed to high levels of solvents (336). Full-time firefighters have been reported to be at increased risk of infertility due to their occupational exposure to several endocrine-disrupting solvents, such as flame retardants and PAHs (337). Of special interest is their exposure to PFAS as the main constituent of aqueous film-forming firefighting foams (338). A human stem cell-based model of spermatogenesis showed that PFAS lower the expression of markers for spermatogonia and primary spermatocytes without directly affecting germ cell viability, suggesting potential long-term effects on male fertility through the depletion of the spermatogonial stem cell pool and abnormalities in primary spermatocytes (339). Additionally, ethylene glycol monomethyl ether (EGME) reduced the expression of spermatocyte-specific genes in rats and mice (340). And ethylene glycol-based chemicals are commonly used as solvents, antifreeze agents, and coolants in commercial and industrial applications.

Epidemiological studies have revealed an increase in fertility disorders, characterized by a higher incidence of lower sperm quality parameters and a greater occurrence of cryptorchidism and hypospadias (341, 342). However, the regulation of spermatogenesis involves numerous and complex underlying biochemical processes that contribute to its dysfunction, including the developmental age of exposure, short- and long-term effects, dosage methods, and dose-related responses. While these EDCs may result in impairments in semen parameters, irrefutable evidence linking these EDCs to the known male fertility decline is elusive. This is partly due to the complexity of conducting longitudinal studies needed to establish causality and the ethical concerns associated with conducting prospective clinical trials involving human exposures. Furthermore, only a limited number of human studies have been conducted to assess the potential long-term or epigenetic effects of low-dose exposures on future generations. Given the current gap in understanding how the environment affects male fertility, it is imperative to minimize exposure to these EDCs and, where feasible, implement prospective cohort studies to clarify the adverse effects on male reproductive health.

#### EDCs affect testicular descent into the scrotal position leading to cryptorchidism

Cryptorchidism, a condition characterized by undescended testes, can significantly impair germ cells and reduce fertility, while also increasing the risk of testicular torsion, hernia, and malignancy. Moreover, it has been a recognized maternal and perinatal risk factor, which can lead to premature birth, low birth weight, and developmental delay of the fetus, thus affecting the growth and development of newborn males.

One prospective analysis revealed a positive association between levels of Bisphenol A (BPA) in maternal sera, placenta, and sera from boys with cryptorchidism. Specifically, higher concentrations of maternal serum BPA during 10-17 weeks of gestation were linked to an increased incidence of cryptorchidism in offspring (343). Additionally, a study focusing on children with cryptorchidism highlighted significantly elevated plasma levels of BPA, BPS, and BPF relative to control subjects (344). Additionally, a retrospective analysis found a heightened occurrence of cryptorchidism in sons of men who were exposed to DES during prenatal development. The transgenerational effects of DES were observed in animal studies as well as in the offspring of women exposed to DES (345). Furthermore, prenatal exposure to environmentally relevant levels of PBDEs leads to testicular dysgenesis in male offspring, which is characterized by decreased anogenital index and testicular organ coefficient, increased incidence of cryptorchidism (346). Moreover, prenatal exposure to atrazine (ATZ), an herbicide used for preventing broadleaf weeds in crops and turf grasses, was shown to induce cryptorchidism and hypospadias in male mouse offspring, potentially through the downregulation of insulin-like 3 (347). In the Norwegian HUMIS study, exposure to PCB-74, PCB-114, PCB-194, and the estrogenic organochlorine pesticide β-hexachlorocyclohexane (β-HCH) may also increase the risk of cryptorchidism (348). However, in the French West, there was no observed association between maternal or cord plasma concentration of Chlordecone, an organochlorine pesticide, and the risk of overall malformations or undescended testes (349). Cryptorchidism displayed spatial heterogeneity, with the main cluster in an area of former coal mining and metallurgic industry in northern France, suggesting environmental factors associated with higher incidence (350). Nevertheless, the research results on the relationship between EDCs and cryptorchidism are not consistent. Other epidemiological studies have found no associations between *in-utero* exposure to PCBs, dioxins, or HCB and cryptorchidism (351).

Several studies noted that some EDCs pass across the placenta in humans throughout pregnancy and different levels of bioaccumulation depending on the fetal organ. Male fetuses appear to be more susceptible to the effects of EDCs compared to female fetuses. While a correlation exists between the occurrence of cryptorchidism and EDCs, a significant association between EDC exposure and the incidence of cryptorchidism has not been definitively established. Consequently, it is crucial to comprehensively evaluate the reproductive toxicity of environmental endocrine disruptors. Further studies are required to explore these specific issues.

#### EDCs interfere with the development of male genitals leading to hypospadias

Hypospadias is one of the most common congenital malformations in the urinary system of children, yet its etiology remains unknown. It is characterized by the urethral opening developing on the ventral side of the penis. Xenobiotic chemicals, including certain drugs and environmental contaminants, especially EDCs, are believed to be the primary factors contributing to the development of hypospadias.

The observation of the intervention of DEHP in female SD rats found that DEHP significantly alters the oxidative balance in male fetal, inducing cryptorchidism and hypospadias by reducing oxidative stress (352). Most retrospective studies reported parental occupational exposure to pesticides was associated with at least a twofold increased risk of hypospadias (353, 354). The VHEMBE birth cohort study examined levels of EDCs in maternal perinatal serum and urine and further correlated this data with the genitourinary examination results of boys in their offspring. The study found that higher prenatal exposure to pyrethroid insecticides was linked to an elevated risk of hypospadias (355). Exposure of pregnant rats to the pesticide pyrifluquinazon (PFQ) and dibutyl phthalate (DBP) during sexual differentiation of the reproductive tract disrupted the androgen signaling pathway and induced hypospadias (356). Nevertheless, slightly increased risks of cryptorchidism were observed in association with all crop acreages near homes during pregnancy, especially for orchards, and no association was observed for hypospadias in French (357). So, residential exposure to pesticide-active ingredients has shown diverse patterns of association with birth defects. Likewise, a specific study investigating the presence of EDCs in breast milk samples of mothers with and without hypospadias-affected baby boys did not find any conclusive evidence linking hypospadias with exposure to POPs (358).

Therefore, it is evident that there are remaining challenges in comprehensively understanding the impact of environmental factors on the occurrence of hypospadias. A vast majority of research claims that being heavily exposed to agricultural pesticides occupies an important position in the pathogenesis of hypospadias, despite the limitation of modest case numbers. These discrepancies could be attributed to individual variations in the absorption, metabolism, or distribution of EDCs. Nonetheless, these studies do provide valuable information on the quantification of exposures. To comprehensively assess the potential combined effects of multiple EDC exposures, the development of multi-exposure models is warranted.

#### EDCs improve the carcinogenic effect of prostate cancer (PC)

The development and maintenance of the prostate gland, like the testes, rely on the local activity of androgens in the body and the regulation of the HPG axis. EDCs produce reproductive toxicity by interfering with the normal function of male hormones. The prostate grows very slowly after sexual maturity and usually causes symptoms only in the severe advanced stage, such as PC. PC, a hormone-dependent cancer, has emerged as a serious global health concern, significantly impacting men’s well-being (359). EDCs represent a major environmental and health issue, as they can disrupt normal prostate functions, stimulate PC growth, and impair immune and neuronal systems.

There are currently some EDCs that can be considered carcinogenic, like dioxin for PC (360). Chronic low-dose exposure to BPA throughout life has been associated with an increased risk of prostate disease. This can occur through interference with genes involved in intraprostatic androgen and estrogen production, as well as other genes implicated in PC development (361–363). Epigenetic markers, such as DNA methylation and histone modifications, may also contribute to these effects (364). BPS, an alternative to BPA, has been found to contribute to the progression of PC by regulating the expression of genes COL1A1 and COL1A2 (365). Furthermore, benzene, a volatile organic compound found in petroleum and crude oil products, has been found to increase the odds of developing PC two-fold, even at low ambient concentrations (366). A forty-year systematic review supports that the most tumorigenic EDC groups were PAEs, heavy metals, and pesticides (367). In a study involving 80 PC patients, frequently detected metabolites of PAEs, such as DEHP, DiBP, and butyl-benzyl (BBzP), were quantified in urine samples using liquid chromatography or electrospray ionization tandem mass spectrometry. The results demonstrated an association between exposure to these metabolites and the occurrence of PC, particularly in abdominally obese men (368). Another exploratory case-control study examining heavy metal toxicities in benign PC estimated Pb and Cd levels using atomic absorption spectrophotometry, demonstrating an increased risk of PC associated with heavy metal toxicity (369). Furthermore, blood cadmium levels exhibited an inverse association with PC, while blood lead, urinary tin, and thallium levels exhibited positive associations (370). A multiple metal analysis about the association between serum heavy metals and PC disclosed that the serum levels of As and Zn increased the risk of PC (371). The risks posed by exposure to these metals are particularly pronounced in PC patients compared to other EDCs. Another study examined the combined effects of pesticides and established PC loci, providing evidence of potential mechanisms through which pesticides may heighten the risk of PC (372). A prospective nested case-control study of serum concentrations of each of the PFAS and the risk of aggressive PC shows that serum concentrations of PFAS did not establish an association with an increased risk of aggressive PC (373). Despite this, *in vitro* experiments verified the promotive role of PFOA and perfluorononanoic acid (PFNA) in the growth of prostate cancer cells (374). Additionally, the cytotoxicity and proliferation of a prostate cancer cell line exposed to various dilutions of PFOA and PCB 153 were monitored, and the results suggest that even at picomolar concentrations, PFOA and PCB153 can increase the proliferation of PC (375).

The aforementioned research provided novel insights into understanding the role of some EDCs in PC and brought attention to the environmental association with male reproductive system cancer risks and progression. Multiple findings have highlighted that even low-concentration exposure to various types of EDCs can induce distinct effects on PC cells, emphasizing the importance of investigating the impact of EDC compounds across all stages of the disease. The disruption of HPG axis secretion arising from EDCs exposure may represent a significant step in the aberrant development leading to PC, while the combined effects of EDC mixtures augment the risk of PC beyond that attributed to individual EDC exposures. Consequently, more prospective studies should compensate for the limitations of mechanistic experiments and incorporate comparable toxicological data.

#### EDCs disrupt the homeostasis of testicular cells leading to testicular cancer (TC)

TC is one of the most common malignancies in young men though the pathogenesis is not completely clear. Cryptorchidism, hypospadias, oligospermia, genetic factors, and EDCs are recognized as high-risk factors for TC. The dysfunction of HPG axis caused by EDCs, resulting in the change of male hormone levels, is the direct cause of cryptorchidism malignant transformation, and may also be the cause of TC. Findings from a systematic review and meta-analysis consistently demonstrate that maternal EDCs exposure, as opposed to postnatal exposure in adult males, is associated with an increased risk of TC (5).

Emerging evidence supported the link between male reproductive system diseases and tumors, especially male infertile and TC. Epidemiology has confirmed the likelihood of illness of TC has increased by more than 60% in infertile men (376). Furthermore, EDCs such as PFOS negatively impact testis-specific biomarkers insulin-like peptide 3 and androstenedione (377). Glyphosate formulations are widely used as a non-selective systemic herbicide and have been shown to affect testicular weight in male guinea pigs, severely impairing testicular growth performance and inducing reproductive toxicity (378). A 20-year ecological study of TC carried out in Brazil manifested that fungicides and insecticides increased age-standardized mortality rates (379). Long-term exposure to Cd and Pb compounds, known to induce acute reproductive toxicity (380), has been associated with significantly higher blood Cd levels in patients with TC, highlighting the potential role of Cd as an important parameter in TC prediction (381). Animal experiments have also demonstrated that the severity of testes injury increases with increasing Cd concentration, which can potentially impact testosterone synthesis (382). In another study, neonatal exposure to EDCs resulted in infertility and testicular tumors in 65% of DES-treated mice (383). Moreover, studies on the mechanism by which EDCs promote the development of TC cells have shown that the connection and non-connection functions of Cx43 and MAPK signaling pathways may be involved in the process of EDCs disrupting the balance of TC cell growth *in vivo* (384).

Although only certain studies have reported an increased risk for TC upon EDCs exposure and the mechanism of action is still ambiguous, the role of EDCs as a threat to male reproductive health cannot be ignored. Exposure to increased EDCs can disrupt androgen expression, disrupt hormone receptor binding, lead to HPG axis imbalance, interfere with steroidogenesis and hormonal metabolism, or modify epigenetic mechanisms, all of which can lead to TDS and other reproductive system cancer diseases. The chronic exposition of cancer cells to EDCs should be considered as a potential obstacle in cancer therapy as many studies showed that exposure to these compounds promotes radio- and chemo-resistance. Despite ongoing research, the precise action modes of certain EDCs in hormone-sensitive cancers and their effects on tumor growth and metastases, particularly in humans, remain poorly understood. Given the wide prevalence and extensive use of EDCs, continued assessment of their long-term reproductive health effects, particularly in carcinogenesis, is essential to eradicate the worst of them and to sensitize the population to minimize their use.

## Conclusion and future prospective

**Figure 4:**
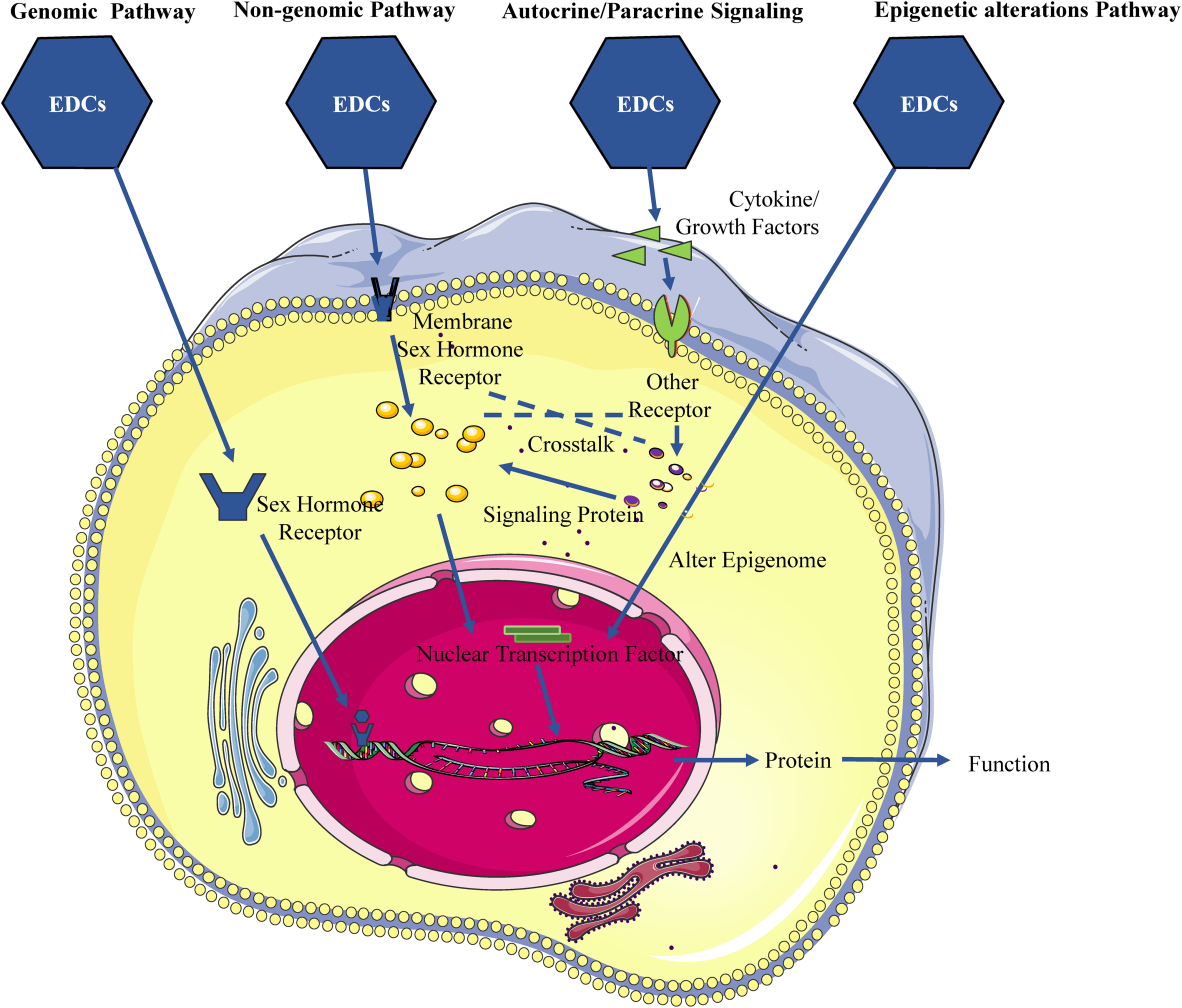
Molecular mechanisms of EDCs-genomic pathways, non-genomic pathways, autocrine/paracrine signaling and epigenetic alterations.

The global health impact of EDCs has become a major public problem, initially and primarily focused on their actions through the HPG axis to induce hormone-like effects. However, research now clearly demonstrates that the mechanisms of action of EDCs are far more diverse. The mechanisms by which EDCs mediate specific cellular functions (e.g., cell growth, proliferation, differentiation, apoptosis and carcinogenesis) lead to the occurrence and development of the above-mentioned reproductive system-related diseases through various common pathways. These pathways involve the genomic regulation of target gene transcription, the non-genomic rapid signal transduction mediated by membrane-bound ERs or other receptors, the autocrine or paracrine signaling pathways involving growth factors and cytokines, and epigenetic alterations majorly in the form of DNA methylation, histone modifications (**Figure 4**). This broader understanding of their actions not only implicates reproductive toxicity but also highlights their potent carcinogenic and cancer-promoting effects. EDCs exist in various forms and are widespread in numerous products. Certain substances can enter the body through inhalation and dietary intake, exerting impacts on the reproductive system and significantly affecting reproductive health, potentially leading to UFs, EMs, PCOS, DOR, POI, infertile, BC, EC, OC, CC, cryptorchidism, hypospadias, PC and TC. In light of the recent reclassification of certain EDCs as human carcinogens, further investigation into the association between the aforementioned outcomes and reproductive system diseases should be conducted with larger sample sizes.

**Supplementary Table 1** provides detailed information on studies that demonstrate the association and mechanism of action between EDCs exposure and reproductive health. The complex feedback mechanisms involved in the hormone-like or anti-hormone-like activity of EDCs can be detected through various methods, such as ligand binding assay, transcriptional assay, protein assay, cell assay, animal assay, and signal pathway analysis. As a result, differences arise in defining pathogenic concentration dependence, agonist or antagonist properties, and ligand or pathway responses, leading to potentially contradictory research findings regarding the same EDCs. A quick and cost-effective solution for preliminary hazard assessment of chemicals that do not undergo animal testing, to identify which chemicals can act as an EDC is a significant advantage and a practical necessity.

Notwithstanding the fact that multiple social and structural determinants of health contribute to the marked racial-, ethnic-, and gender-based disparities in endocrine, EDCs exposure undoubtedly generally tend to accumulate and resist degradation, ampling the risk of endocrine diseases and reducing the quality of life. Vulnerable populations carry a disproportionate burden of multiple reproductive disorders and their associated comorbidities and medical costs. Addressing these concerns will necessitate political determination to develop and implement different remediation policies based on different degrees of degradation levels to limit the use of these harmful EDCs. The incorporate occupational and environmental history-taking into medical practice would facilitate the diagnosis and treatment of reproductive system diseases. Nonetheless, implementing personalized education programs, modifying unhealthy dietary habits, choosing fresh food instead of processed food, washing fruits and vegetables to remove residual pesticides, avoiding heating or microwaving food in plastic containers, minimizing exposure to EDC chemicals, and taking relevant protective measures in occupations with high exposure are fundamental strategies to reduce the risk of diseases related to the reproductive system. These measures are crucial in minimizing exposure during the development of female pregnancy and male sexual organs, preventing the accumulation of EDCs in the body, and mitigating the genetic effects of intergenerational relationships.

The existing research on EDCs and their effects on reproductive health still has several areas for improvement. The aforementioned studies have several limitations, primarily stemming from their retrospective nature and lack of specific focus on investigating the association between EDCs half-life and reproductive system diseases. To comprehensively characterize the effects of individual exposures and their combinations, future research should aim to collect multiple exposure data and consider the presence of EDC mixtures, as stronger associations have been observed with the development of cancer for mixtures compared to individual chemicals (385). Additionally, efforts should be directed toward identifying potential etiologic agents or mixtures of compounds that may act synergistically or through specific mechanistic pathways. It is crucial to capture exposure data during critical developmental periods to shed light on the relationship between exposure and disease, with a particular emphasis on precaution, prevention, and potential multigenerational effects. Indeed, in different species and at different times of the life cycle, an EDC can have different effects on different cell or tissue types. Large-scale, well-designed longitudinal prospective research is needed to gather information from diverse populations worldwide and conduct more robust epidemiological studies to confirm the mechanisms of action and the importance of critical exposure windows, in order to fully comprehend the impact of EDCs on reproductive health.

## Author contributions

JP: Writing – original draft. PL: Investigation, Writing – review & editing. XY: Investigation, Writing – review & editing. ZZ: Writing – review & editing. JL: Conceptualization, Writing – review & editing.
