# Supplementary material for: The adverse role of endocrine disrupting chemicals in the reproductive system
Source: Front Endocrinol (Lausanne). 2024 Jan 17;14:1324993. doi: 10.3389/fendo.2023.1324993 (PMC10832042; doi:10.3389/fendo.2023.1324993)
Supplement: Supplementary file 1 [file Table_1.doc]

**Supplementary Table 1. The association between EDCs exposure and reproductive health**

| **Type of EDCs** | **Source of exposure** | **Model** | | **Dosage** | **Exposure time** | **Main actions** | **Endpoints** | **References** |
| --- | --- | --- | --- | --- | --- | --- | --- | --- |
| **BPA** | epoxy resin linings of metal-based food, beverage cans, thermal paper, medical equipment, toys, electronics, and water pipes | Human:1693 Black women ages 23–34 years |  | | 60 months | environment; lifestyle | uterine fibroids **+** | Bethea T N et al.(2020) |
|  |  | human uterine leiomyoma cells | 10^-6^-10μM; 100-200μM | | 24 h; 48 h; 72 h | Src, EGFR, Ras, and MAPK signaling pathways **↑** | uterine fibroids **+** | Yu L et al.(2019) |
|  |  | human uterine leiomyoma cells | 1 μmol/L; 10μmol/L | | 72 h | GPR30-EGFR and MAPK/ERK/c-fos signaling pathway **↑** | uterine fibroids **+** | Li Z et al.(2019) |
|  |  | human uterine leiomyoma cells | 10 μmol/L | | 24 h; 48 h; 72 h | TGF-β signaling pathway **↑** | uterine fibroids **+** | Shen Y et al.(2018) |
|  |  | human primary uterine leiomyoma cells | 10 μmol/L | | 48 h | PI3K-AKT signaling pathway **↑** | uterine fibroids **+** | Li Z et al.(2021) |
| **BPA** |  | human primary uterine leiomyoma cells | 10μmol/L | | 48 h | ITGA2 ↑ PI3K/AKT signaling pathway **↑** | uterine fibroids **+** | Li Z et al.(2022) |
|  |  | Human:754 Black women aged 23-35 years |  | | 60 months | environment; lifestyle | uterine fibroids **+** | Wesselink A K et al.(2021) |
|  |  | human endometrial stromal cell lines | 1,10,100 nM | | 48 h | MMP 2 and MMP9 **↑** | endometriosis + | Wen X et al.(2020) |
|  |  | Human: women aged 18-45 |  | |  | environment | endometriosis **×** | Moreira Fernandez M A et al.(2019) |
|  |  | Human:700 women |  | |  | environment | endometriosis + | Lee A W et al.(2022) |
|  |  | Human:124 women |  | |  | oxidative stress | endometriosis + | Peinado F M et al.(2020) |
| **BPA** |  | human normal endometrial stromal cells | 1,10, 100 nM | | 48 h | WDR5/TET2-mediated epigenetic pathway ↑ | endometriosis + | Xue W et al.(2021) |
|  |  | Animal: female mice | 3,30,90mg/kg/day | | 6 weeks | hypothalamic gonadal axis | endometriosis + | Jones R L et al.(2018) |
|  |  | Animal:CD1 mice | 50 μg /kg/day | | 2 weeks | collagen ↑ F4/80 positive macrophage ↑ | uterine pathology + | Kendziorski JA et al.(2015) |
|  |  | human endometrial stromal cells | 10–1000 pmol | |  | oxidative stress ↑ inflammatory signals ↑ | endometrium-related disorders + | Cho Y J et al.(2018) |
|  |  | Human:62 girls with disease and 33 controls, age 12-18 years |  | |  | neuroendocrine; reproductive; metabolic regulation | polycystic ovary syndrome + | Akgül S et al.(2019) |
|  |  | Human:199 women with disease and 158 control |  | |  | environment; lifestyle | polycystic ovary syndrome + | Jurewicz J et al.(2021) |
| **BPA** |  | Human:29 women |  | |  | metabolic ↑ | polycystic ovary syndrome + | Milanović M et al.(2020) |
|  |  | Human:321 women with disease and 412 controls |  | |  | reproductive toxicity ↑ | polycystic ovary syndrome + | Zhan W et al.(2023) |
|  |  | Animal: female adult rats | 0.001, 0.1mg/kg  bw | | 90 days | folliculogenesis; CDKN2A and ROS levels↑ | polycystic ovary syndrome + | Prabhu N B et al.(2022) |
|  |  | Human: women with disease, age 18-45 years |  | |  | steroid hormone biosynthesis; metabolism | polycystic ovary syndrome + | Prabhu N B et al.(2023) |
|  |  | Animal: Wistar rats | 10 ppm/d | | 180 days | metabolic; insulin resistance | polycystic ovary syndrome + | Dabeer S et al.(2020) |
|  |  | human ovarian granulosa KGN cells | 0.5, 5, 50, 500 μg/L | | 6 h | hormone synthesis; FSHR/GS/AC signaling pathway **↓** | polycystic ovary syndrome + | Shi J et al.(2021) |
| **BPA** |  | female medaka | 10 μg/L | | 8 h post fertilization-d 15 after fertilization | cancerous pathway **↑** arginine-proline metabolism **↑** | polycystic ovary syndrome + | Chakraborty S et al.(2023) |
|  |  | Animal: neonatal female rats | 50 μg/kg/day | | Postnatal 1-16 | lipid metabolism **↑** steroidogenesis **↑** | polycystic ovary syndrome + | Yang Z et al.(2019) |
|  |  | Human:111 women |  | |  | environment | diminished ovarian reserve + | Zhang N et al.(2023) |
|  |  | Human:307 Korean reproductive-aged women |  | |  | AMH **↑** | diminished ovarian reserve + | Park S Y et al.(2021) |
|  |  | Human:159 women disease and 186 controls |  | |  | LH **↑** | premature ovarian insufficiency **×** | Li C et al.(2021) |
|  |  | Animal: mice; granulosa cells | 10 μg/kg/day | | post coitus day 12.5-18.5 | endoplasmic reticulum stress ↑ | premature ovarian insufficiency **+** | Chen W et al.(2023) |
| **BPA** |  | Human:30 women disease and 30 controls |  | |  | environment; lifestyle | premature ovarian insufficiency **+** | Özel Ş et al.(2019) |
|  |  | human granulosa cell line | 0, 0.1, 1, 10, 100 μM | | 12 h; 24 h | ROS/Ca^2+^-ASK1-JNK signaling pathway ↑ | female infertility **+** | Huang M et al.(2021) |
|  |  | Human: 857 women of reproductive age |  | |  | environment | female infertility **+** | Zhan W et al.(2022) |
|  |  | Animal:CD-1 (ICR) male mice | 50 mg/kg day, bw/ | | 6 weeks | epigenetic modification | female infertility **+** | Ryu D Y et al.(2022) |
|  |  | bovine cumulus-oocyte complexes | 1 fM - 50 μM | | 24 h | spindle abnormalities; chromosome misalignment | female infertility **+** | Campen K A et al.(2018) |
|  |  | Animal: female mice; human granulosa cell line | 1, 10, 100 μg/kg; 1, 10, 100 nM | | 2 weeks; 24,48, 72h | AMPK/mTOR/ULK1 signaling pathway **↑** | female infertility **+** | Lin M et al.(2021) |
| **BPA** |  | Human:450 women aged 24-44 |  | |  | environment | female infertility **×** | Radwan P et al.(2020) |
|  |  | Human:203 couples |  | |  | preconception exposure | preterm **+** | Zhang Y et al.(2021) |
|  |  | Animal: female SD rats | 0,25,250,2500,25000/kg /day, bw | | gestational 6-1 years old | gene expression | endometrial cancer **+** | Leung Y K et al.(2020) |
|  |  | Animal:C57BL6 mice | 9μg/kg/day | | 60 days | steroid hormone signaling **↓** | uterine hyperplasia and cancer **+** | Neff A M et al.(2019) |
|  |  | primary ovarian cell line | 10,100 nM | |  | biological pathways | ovarian cancer **+** | Zahra A et al.(2022) |
|  |  | Human: 45 women |  | |  | environment | cervical cancer **+** | Medellín-Garibay S E et al.(2023) |
| **BPA** |  | Human: 135 men |  | |  | environment | female infertility **+** | Mantzouki C et al.(2019) |
|  |  | Human: 52 women |  | |  | environment | breast cancer **+** | Keshavarz-Maleki R et al.(2021) |
|  |  | 7820 breast cancer cases |  | |  | environment | breast cancer **×** | Liu G et al.(2021) |
|  |  | human breast cancer cells (MCF-7) |  | |  | mitochondrial biogenesis | breast cancer **+** | Ansari M I et al.(2022) |
|  |  | human breast cancer cells (MCF-7) | 50,100,200 nM;1μM | | 200 days | mitochondrial biogenesis;  epithelial-mesenchymal plasticity | breast cancer **+** | Ansari M I et al.(2022) |
|  |  | Animal: zebrafish | 1 μg/g | | 3–8 months | environment | infertility **+** | Mostari M H et al.(2022) |
| **BPA** |  | Animal: adult male mice | 0.2,20,200 μg/ml | | 2 months | diet | male infertility **+** | Liu X et al.(2021) |
|  |  | Animal: adult male mice | 3,30,300  mg/kg/day | | 5 weeks | germ cell apoptosis | male infertility **+** | Jiang X et al.(2018) |
|  |  | Human: men with or without azoospermatism |  | |  | MicroRNAs Levels | male infertility **+** | Palak E et al.(2021) |
|  |  | Human: 157 male factory workers |  | |  | DNA hydroxymethylation | male infertility **+** | Song X et al.(2019) |
|  |  | Human: reproductive-aged Chinese men |  | |  | reproductive hormones | male infertility | Zeng J Y et al.(2022a) |
|  |  | Animal: male mice | 50mg/kg/day,bw | | 6 weeks | epigenetic modification | male infertility **+** | Ryu D Y et al.(2022) |
| **BPA** |  | Human: fertile men |  | |  | sperm concentration; sperm swing characteristics | male infertility **+** | Ji H et al.(2018) |
|  |  | Human: 158 healthy men aged 18-23 |  | |  | sperm DNA fragmentation | male infertility **+** | Kiwitt-Cárdenas J et al.(2021) |
|  |  | Human: 80 men |  | |  | environment | male infertility | Mantzouki C et al.(2019) |
|  |  | Human: 158 male factory workers |  | |  | DNA hydroxymethylation | male infertility | Tian Y et al.(2018) |
|  |  | Human: 146 couples undergoing in vitro fertilization |  | |  | environment | male infertility **×** | Kim H K et al.(2021) |
|  |  | Human: 556 men aged 18-20 |  | |  | environment | male infertility **×** | Benson T E et al.(2021) |
| **BPA** |  | Human: 105 men in a fertility clinic |  | |  | environment | male infertility **×** | Caporossi L et al.(2020) |
|  |  | Human: young Danish men |  | |  | environment | male infertility **×** | Joensen U N et al.(2018) |
|  |  | Human:98 children with cryptorchidism and 19 controls |  | |  | environment | Cryptorchidism **+** | Komarowska M D et al.(2021) |
|  |  | Animal: male and female Wistar rats | 2.5 μg/kg bw/d | | gestational 12-parturition | genetic modification | prostate cancer **+** | Sánchez P et al.(2022) |
|  |  | Human:4812 participants |  | |  | environment | prostate cancer **+** | Salamanca-Fernández E et al.(2021) |
| **PAEs** | cosmetics, glues, certain detergents, medical products, enteric coating | Human: women aged 20-54 |  | |  | environment; diet | uterine leiomyomata **-**  endometriosis **-** | Zhang Y et al.(2021) |
| **PAEs** |  | Human:57 premenopausal women |  | |  | environment | uterine fibroids + | Zota A R et al.(2019) |
|  |  | Human:130 women |  | |  | DNA damage | uterine fibroids + endometriosis + | Zhang M et al.(2023) |
|  |  | primary leiomyoma and smooth muscle cells | 1.6μM | | 48, 72 h | tryptophan-kynurenine-AHR pathway **↑** | uterine fibroids + | Iizuka T et al.(2022) |
|  |  | Human: women aged 45-54 |  | |  | lifestyle | uterine fibroids + | Pacyga D C et al.(2022) |
|  |  | Human:512 women of reproductive age |  | |  | environment | uterine fibroids + | Lee J et al.(2020) |
|  |  | Human: black women aged 23-35 |  | | 20, 60months | environment; lifestyle | uterine fibroids | Fruh V et al.(2021) |
| **PAEs** |  | Animal: five-week-old female NOD/SCID mice | 500 mg/kg/day | | 8 weeks | VEGF, collagen↑ AKT phosphorylation ↑ | uterine fibroids + | Kim H J et al.(2021) |
|  |  | human leiomyoma cells | 0-6μM | | 24, 48 h | HIF-1α **↑** COX-2 **↑** | uterine fibroids + | Kim J H (2018) |
|  |  | Animal:54 SD rats | 0, 5, 50, 100, 250, 500, 1,000, 2000, 3000 mg/kg/day | | 2 weeks | steroid biosynthesis pathway | endometriosis + | Yi H et al.(2023) |
|  |  | primary endometrial stromal cells and epithelial cells | 0.1,1,10µM | | 72 h | EnSC invasiveness ↑ | endometriosis + | Gonzalez-Martin R et al.(2022) |
|  |  | human granulosa cells | 67.5, 250, 500μg/mL | | 24 h | gene expression; mitochondrial membrane potential **↓** | endometriosis + | Chou Y C et al.(2022) |
| **PAEs** |  | Animal:C57BL/6 female mice | 0.5 mg/kg/day bw | | 10 days; 4 weeks | CD44 **↑** | endometriosis + | Sharma P et al.(2021) |
|  |  | Human: endometriosis patients aged 20-43 |  | |  | aldo-keto reductases**↑** | endometriosis + | Kim Y et al.(2017) |
|  |  | Animal: NOD/SCID mouse; endometrial cell | 500, 1000 mg/kg/day;  10^-6^,10^-8^M | | 2 weeks; 24 h | MMP-2, MMP-9 ↑ Erk phosphorylation↑ | endometriosis + | Kim S H et al.(2015) |
|  |  | Human:124 adolescents |  | |  | metabolic disturbances | polycystic ovary syndrome **+** | Akın L et al.(2020) |
|  |  | Human:599 Saudi women |  | |  | environment | polycystic ovary syndrome **+** | Al-Saleh I (2022) |
| **PAEs** |  | Human:60 women |  | |  | obesity, glucose and lipid impairment | polycystic ovary syndrome **+** | Milankov A et al.(2023) |
|  |  | Human:271 women |  | |  | environment | ovarian reserve **×** | Génard-Walton M et al.(2023) |
|  |  | Human:264 childbearing-age women |  | |  | COR **↓** CORT ↓ | diminished ovarian reserve **+** | Li Y et al.(2023) |
|  |  | Animal: ICR female mice; the ovarian granulosa cell line KGN | 2g/kg/day; 25μM | | 8 days; 12 h | SLC39A5/NF-κB/NLRP3 axis**↑** | decrease ovarian reserve | Sun J et al.(2023) |
|  |  |  |  | |  | tumor necrosis factor | premature ovarian insufficiency | Zhang F L et al.(2021) |
|  |  | Animal: pregnant CD-1 mice | 20 μg/kg/day;  500mg/kg/day | | gestational 10 to birth | biomarkers of reproductive aging | premature ovarian insufficiency | Brehm E et al.(2020) |
| **PAEs** |  | Animal: female SD rats |  | | 6 weeks | primary follicles **↓** | premature ovarian failure **+** | Tran D N et al.(2018) |
|  |  | Human:173 with disease and 246 controls |  | |  | estradiol/FSH ratio | premature ovarian failure **+** | Cao M et al.(2020) |
|  |  | Human:186 women under 43 years of age |  | |  | work activities; lifestyle habits | female infertility | Caporossi L et al.(2021) |
|  |  | Human:325 females |  | |  | environment; lifestyle | female infertility + | Abdo N et al.(2023) |
|  |  | Human:333 women |  | |  | environment | female infertility + | Bellavia A et al.(2023) |
|  |  | Human:138 women |  | |  | AMH ↓ | female infertility **+** premature ovarian failure **+** | Sacha C R et al.(2021) |
| **PAEs** |  | data from surveys |  | |  | environment | female infertility + | Trnka B et al.(2021) |
|  |  | Human:210 couples |  | |  | environment | female infertility + | Mínguez-Alarcón L et al.(2021) |
|  |  | Human:194 women |  | |  | reproductive hormones | female infertility | Du Y et al.(2019) |
|  |  | Animal: wistar rat | 30,60  mg/kg/day bw | | gestational 7 -postnatal 22 | anti-androgenic | female infertility | Boberg J et al.(2021) |
|  |  | Human: postmenopausal women |  | |  | environment | endometrial cancer **+** | Sarink D et al.(2021) |
|  |  | Human:1308 women |  | |  | environment | breast cancer **+** | Parada H, Jr. et al.(2018) |
| **PAEs** |  | Human:102 women |  | |  | environment | breast cancer **+** | Segovia-Mendoza M et al.(2022) |
|  |  | human breast cancer cells (MCF-7) | 10^−6^M | | 1 h | estrogenicity | breast cancer **+** | Fiocchetti M et al.(2021) |
|  |  | human breast cancer cells (MCF-7) | 1, 10, 100 µM | | 24 h | aryl hydrocarbon receptor **↑** | breast cancer **+** | Shan A et al.(2020) |
|  |  | Human:66 women |  | |  | ADAM33 **↑** | breast cancer **+** | Yang P J et al.(2018) |
|  |  | breast tissues |  | |  | estrogenicity | breast cancer **+** | Amin M M et al.(2019) |
|  |  | Human: males with infertility |  | |  | reproductive hormones;  semen quality | male infertility | Wang B et al.(2020) |
| **PAEs** |  | Human: 339 males |  | |  | semen quality | male infertility | Smarr M M et al.(2018) |
|  |  | Human: 210 couples undergoing in vitro fertilization |  | |  | environment | infertility **+** | Mínguez-Alarcón L et al.(2021) |
|  |  | Human: 111 men from an infertility clinic |  | |  | spermatozoa apoptosis | male infertility | Xiong F et al.(2022) |
|  |  | Human: 100 idiopathic infertile males |  | |  | sperm acrosomal reaction | male infertility | Cosci I et al.(2022) |
|  |  | mouse spermatogonia-derived cells | 50,100,200,  400µM | | 48 h | telomere dysfunction | male infertility | Zhou F et al.(2023) |
|  |  | Animal: Swiss albino mice | 1,10 mg/kg,bw | | 3 months | apoptosis | male infertility | Mondal S et al.(2019) |
| **PAEs** |  | Animal: pregnant SD female rats | 750 mg/kg/day | | gestational days 14-21 | oxidative stress | testicular dysgenesis syndrome + | Li Q et al.(2022) |
| **Dioxin** | waste incineration | Human: women |  | |  | environment | breast cancer **+** | Vopham T et al.(2020) |
|  |  | Human: 1145 women |  | |  | environment | breast cancer **-** | Danjou A M N et al.(2019 |
| **PBDEs** | flame retardants | human breast cancer cells (MCF-7) |  | |  | nuclear receptor signaling pathways | breast cancer **+** | Kanaya N et al.(2019) |
|  |  | Animal: breast cancer nude mice | 0, 1, 10, 100 mg/kg bw | | 6 weeks | cytokines and receptor | breast cancer **+** | Wei J et al.(2020) |
|  |  | Human:374 women |  | |  | environment | breast cancer **+** | He Y et al.(2018) |
| **PBDEs** |  | Human:50 women |  | |  | environment | breast cancer **+** | Li A J et al.(2019) |
|  |  | Animal: pregnant ICR mice | 0.2,2,20 mg/kg/day | | gestational days 1-21 | apoptosis **↑** | cryptorchidism **+** | Zhao T et al.(2022) |
| **OPEs** | flame retardants; lubricants; plasticizers | Human: Korean women aged 20-49 |  | |  | estrogenic activity | uterine fibroids **+** | Lee G et al.(2020) |
| **DES** | drug | Animal: mouse pups | 2 µg/pup/day | | postnatal days 5–7; 1–5 respectively | stem cells | uteropathies | Singh P et al.(2022) |
|  |  | Animal: female Eker rats | 11μg/rat/day | | days 10-12 after birth | epigenetic modulation | uterine fibroids **+** | Yang Q W et al.(2023) |
|  |  | Animal: female Eker rats | 10μg/rat/day | | days 10-12 after birth | developmental reprogramming | uterine fibroids **+** | Yang Q et al.(2023) |
| **DES** |  | Animal: 5-month-old Eker rats | 10μg/day | | postnatal 10-12 | TGF-β pathway **↑** | uterine fibroids **+** | Bariani M V et al.(2023) |
|  |  | Animal: female newborn Eker rats | 10μg/day | | days 10-12 after birth | DNA damage | uterine fibroids **+** | Elkafas H et al.(2020) |
|  |  | Animal: female Wistar albino rats | 1 mg/kg/day bw | | 20 days | erratic estrus cycle; aberrant hormone levels | endometriosis **+** | Krishnamoorthy S P et al.(2022) |
|  |  | Human ovarian cortical tissue | 10^-10^ - 10^-6^ M | | 6 days | environment | female infertility | Li T et al.(2023) |
|  |  | Animal: adult Swiss and FVB mice | 2 µg/day | | 5 days | epigenetic | female infertility; testicular tumors | Kaushik A et al.(2023) |
| **HCB** | a seed-mixing fungicide; fireworks colorant | Human endometrial stromal cells; endometrial stromal cells |  | |  | aromatase **↑** | endometriosis **+** | Chiappini F et al.(2022) |
| **HCB** |  | Animal: female rats | 1, 10, 100 mg/kg bw | | 30 days | VEGF ↑ COX-2 ↑ AhR ↑ | endometriosis **+** | Chiappini F et al.(2019) |
|  |  | Human:185 women aged 21-43 |  | |  | environment; lifestyle | female infertility **+** | Björvang R D et al.(2022) |
| **PCBs** | dielectric or flame-retardant plasticizers of e-waste material | endometrial cells; endometriosis mouse model | 0.3,3,30μM; 0.03,0.3, 5,50 mg/kg | | Cell:48 h; Animal:2 days | inflammation interactions | endometriosis **+** | Huang Q et al.(2017) |
|  |  | Human: women aged 20-38 |  | |  | endometrial thickness**↓** retrieved oocytes rate↓ fertilization rate↓ | female infertility **+** | Al-Hussaini T K et al.(2018) |
|  |  | Human:374 Chiese women |  | |  | environment | breast cancer **+** | Huang W et al.(2019) |
|  |  | Human:2606 mother-infant pairs |  | |  | environment | cryptorchidism **+** | Desalegn A A et al.(2021) |
| **TBT** | a reducing reagent; organometallic xenobiotic | Animal: neonatal female rats | 10 ng/kg/day; 100 ng/kg/day | | postnatal day 1-16 | lipid metabolism; steroidogenesis pathways | polycystic ovarian syndrome **+** | Yang Z et al.(2019) |
|  |  | Animal: male Syrian hamsters | 50,100,150  ppm/kg/day | | 65 days | steroidogenesis | female infertility | Kanimozhi V et al.(2018) |
| **DDT** | organochlorine pesticides | Human:178 Chiese women |  | |  | androgen catabolism | polycystic ovarian syndrome **+** | Guo Z et al.(2017) |
|  |  | Human:585 women aged 50-54 |  | |  | environment | breast cancer **+** | Cohn B A et al.(2019) |
| **DDE** | persistent organic pollutant | Human:138 cases and 151 controls |  | |  | environment | diminished ovarian reserve **+** | Génard-Walton M et al.(2023) |
|  |  | Human:748 women |  | |  | ER | breast cancer **+** | Parada H, Jr. et al.(2019b) |
| **DDE** |  | Human: highly exposed African American |  | |  | environment | breast cancer **-** | Rusiecki J A et al.(2020 |
|  |  | Human:374 Chiese women |  | |  | environment | breast cancer **+** | Huang W et al.(2019) |
| **PFAS** | fluorinated organic compounds; surfactants; stain repellents; drinking water; foods; food packaging materials | Human: 29106 females | above 10000 ng/L | |  | environment | polycystic ovarian syndrome **+** | Hammarstrand S et al.(2021) |
|  |  | Human:180 with disease and 187 controls |  | |  | environment | polycystic ovarian syndrome **+** | Wang W et al.(2019) |
|  |  | Human:366 women with disease and 577 controls |  | |  | environment | polycystic ovarian syndrome **+** | Zhan W et al.(2023) |
|  |  | Human:1185 women |  | |  | lifestyle | female infertility | Hallberg I et al.(2023) |
| **PFAS** |  | Human:382 women |  | |  | environment | female infertility **+** | Cohen N J et al.(2023) |
|  |  | Human:936 Chinese couples |  | |  | environment | female infertility **+** | Luo K et al.(2022) |
|  |  | Human: male |  | |  | environment | prostate cancer **+** | Chuang S C et al.(2020) |
|  |  | Human:388 French women |  | |  | environment | breast cancer **+** | Mancini F R et al.(2020) |
|  |  | Human:1760 French women |  | |  | environment | breast cancer **×** | Hurley S et al.(2018) |
| **PFOA** | water and stain resistant coatings for carpets, textiles; non-stick cookware; fire-fighting foams; food packaging | Human:75 patients |  | |  | metabolism | diminished ovarian reserve **+** | Shen H et al.(2023) |
| **PFOA** |  | Animal: female mice | 2.5 mg/kg | | 15 days | metabolism | ovotoxicity | González-Alvarez M E & Keating A F (2023) |
|  |  | cancer cell lines |  | |  | Akt/mTORC1 ↑ PlexinD1 ↑ | breast cancer; prostate cancer | Charazac A et al.(2022) |
|  |  | Animal: female rats | 333 µL/100g, bw | | 21 days | hormones; phenotypic;  transcriptomic | breast cancer **+** | Su Y et al.(2022) |
|  |  | human breast epithelial cells | 100 nM  -1 mM | |  | proliferation **↑**  migration **↑**  invasion **↑** | breast cancer **+** | Pierozan P et al.(2018) |
|  |  | Animal: female rats |  | |  | estrogen signaling ↑ | breast cancer **+** | Su Y et al.(2022) |
|  |  | male human embryonic  stem cells | 11,25,100µM | | 10 days | spermatogenesis | male infertility | Steves A N et al.(2018) |
| **PFOS** | stain, water, and grease repellents | Human:120 Chinese women |  | |  | environment | primary ovarian insufficiency **+** | Zhang S et al.(2018) |
|  |  | Human:750 women with disease and 750 controls |  | |  | environment | prostate cancer | Rhee J et al.(2023) |
|  |  | male human embryonic  stem cells | 24,48,126µM | | 10 days | spermatogenesis | male infertility | Steves A N et al.(2018) |
| **PP** | antibacterial preservatives | Animal: Balb/c mice; human ovarian granulosa tumor-derived cell line | 100-1000 mg/kg/day; 1-1000 nM | | 21 days; 24 h | environment | polycystic ovary syndrome; female infertility | Jiao L et al.(2021) |
| **Pesticides** | drinking water; food; foodstuffs | Human:172 women with disease and 247 controls |  | |  | environment | primary ovarian insufficiency + | Li C et al.(2018) |
| **Pesticides** |  | Animal: female mice | 6.7, 20,60 μg/kg/day | | gestational days 0.5 to 44 weeks old | environment | primary ovarian insufficiency + | Ma X et al.(2022) |
|  |  | porcine granulosa cells | 25μM; 175μM | |  | oxidative stress; DNA damage | diminished ovarian reserve | Wang W et al.(2018) |
|  |  | Human:157 women with disease and 217 controls |  | |  | environment | primary ovarian insufficiency + | Pan W et al.(2019) |
|  |  | Animal: sexually mature female rats | 6.3, 11.33 mg/kg/day bw | | 14 days | environment | premature ovarian insufficient | Ghosh R et al.(2018) |
|  |  | Human:2680 American women |  | |  | environment | female infertility + | Huang J et al.(2023) |
|  |  | Animal: female rats | 0.01,1  mg/kg/day;  3 days/week | | 100 days | environment | female infertility;  uterine proliferative pathologies | Ramos Nieto M R et al.(2021) |
| **Pesticides** |  | Human:421 men in China |  | |  | testosterone concentrations | male infertility | Zeng J Y et al.(2022b) |
|  |  | Animal: pregnant SD rats | 100 mg/kg, bw | | gestational days 8-15 | epigenetic modification | male infertility | Song Y and Yang L(2018) |
|  |  | Human:387 Chinese men |  | |  | semen quality | male infertility | Miao Y et al.(2022) |
|  |  | human semen of males aged 25-40 |  | |  | lysine glutarylation; mitochondrial dysfunction | male infertility | Yang L et al.(2023) |
|  |  | Human: 159 couples with infertility |  | |  | chromosomal abnormalities | male infertility | Figueroa Z I et al.(2019) |
|  |  | Animal: mature male rats | 1.6 mg/kg | |  | testicular function impairment | male infertility + | Ali Abd El-Rahman H and Omar A R, 2022 |
| **Pesticides** |  | Animal: 47 Swiss albino adult male mice | 0.1,0.2 ml DM/100 ml distilled water | | 20 days | sperm morphology | male infertility + | Sasi S M et al.(2023) |
|  |  | bovine sperm | 10, 15, 25 μg/ml | | 2 h | sperm parameters**↓** | male infertility + | Carranza-Martin A C et al.(2023) |
|  |  | Animal: adult male Wistar rats | 500 mg/kg/day bw | | 65 days | environment | male infertility | Girish B P and Reddy P S (2018) |
|  |  | mouse testicular cells | 0,0.5,1,2, 5 μM | | 24 h | mitochondrial dysfunction | male infertility + | Ham J et al.(2021) |
|  |  | sperm from mature ICR mice | 0.1,1,10,100  μM | | 90 min | sperm parameters**↓** | male infertility + | Bae J W and Kwon W S,2021a |
|  |  | Animal: male C57BL/6 mice | 365/730 mg/kg/day | | 28 days | testicular Sertoli cell damage | male infertility | Sun W et al.(2022) |
| **Pesticides** |  | sperm from mature ICR male mice | 0.1, 1, 10, 100, 300 μM | | 90 min | sperm parameters**↓** | male infertility + | Bae J W and Kwon W S,2020 |
|  |  | mouse testis cell lines | 0,2.5,5,7.5μM | | 24 h | mitochondrial dysfunction | male infertility + | Ham J et al.(2020) |
|  |  | Animal: adult male rabbits | 100 mg/L water | | 2 weeks | spermatogenic and Leydig cell | male infertility + | Alamgir Kobir M et al.(2023) |
|  |  | Animal: adult rats | 5, 9,1.9 mg/kg/day | | five times a week for one month | oxidative stress | male infertility + | Abdel-Razik R K et al.(2021) |
|  |  | Animal: adult rats | 2, 10, 70, 100 mg/kg/day,bw | | gestation day 6 to postnatal day 21 | estrogenic and/or anti-androgenic activity | male infertility + | Pandey N et al.(2021) |
|  |  | frozen bovine semen | 1, 5, 10, 25, 50 μg/mL | |  | DNA methylation | male infertility | Pallotta M M et al.(2019) |
| **Pesticides** |  | testicular cells | 10-50 μM | | 12 h;  24 h | reactive oxygen;  phosphorylation of AMPK | male infertility | Chen R et al.(2018) |
|  |  | Animal: male Wistar rats | 3.0 mg /kg,  bw | | 20 weeks | spermatogenesis | male infertility | Li J et al.(2019) |
|  |  | Human: 100 male groundnut farmers |  | |  | semen quality | male infertility | Lwin T Z et al.(2018) |
|  |  | Animal: Balb/c mice | 5 mg/kg bw | | 31 days | DNA damage;  mutations | infertility + | Sharma A et al.(2022) |
|  |  | sperm from ICR male mice | 0.1, 1, 10, 100 μM | |  | protein tyrosine phosphorylation | male infertility | Bae J W and Kwon W, 2021b |
|  |  | Human: men with oligozoos-permia |  | |  | sperm morphology | male infertility | Alagöz M et al.(2023) |
| **Pesticides** |  | Human: 346 men for conceive |  | |  | semen quality | male infertility | Hu Y et al.(2020) |
|  |  | Animal: ICR mice | 10,100  mg/kg/day | | embryonic day 12.5-16.5 | environment | cryptorchidism **+** hypospadias **+** | Tan H et al.(2021) |
|  |  | Human:219 boys |  | |  | occupational exposure | hypospadias **+** | Das D et al.(2023) |
|  |  | Human:1068 pregnant women |  | |  | environment | cryptorchidism × | Rouget F et al.(2020) |
|  |  | Human: birth of 8766 hypospadias cases |  | |  | environment | hypospadias **+** | Bougnères P et al.(2021) |
|  |  | Human:9281 boys |  | |  | residential exposure | cryptorchidism × | Cognez N et al.(2019) |
| **Pesticides** |  | Human: males |  | |  | environment | testicular cancer **×** | Franco A P S et al.(2023) |
|  |  | Animal:40 sexually mature male guinea | 0, 186, 280,560 mg/kg, bw | | 60 days | environment | male infertility **+** | Mutwedu V B et al.(2021) |
|  |  | Human:1162 cases and 2206 controls |  | |  | environment | prostate cancer **+** | Hurwitz L M et al.(2023) |
|  |  | Human: 68 one-year-old boys |  | |  | environment | hypospadias **+** | Bornman R et al.(2022) |
| **TCS** | lipid-soluble, chlorinated aromatic compound as an ingredient in personal care products | Human:109 women |  | |  | environment; diet | diminished ovarian reserve **+** | Mínguez-Alarcón L et al.(2017) |
|  |  | Human:1182 couples who planned to conceive |  | |  | environment | female infertility **+** | Zhu W et al.(2019) |
| **VCD** | flame retardant; antioxidant; plasticizer | Animal: wild-type C57BL/6J female mice | 160 mg/kg/day | | 15 days | environment | premature ovarian failure **+** | Cao L B et al.(2020) |
| **TCDD** | a by-product during organic synthesis and burning |  |  | |  | environment | female infertility + | Rattan S et al.(2017) |
|  |  | human breast cancer cells (MCF-7) | 1, 10, 100 nM | | 24 h | aryl hydrocarbon receptor **↑** | breast cancer **+** | Shan A et al.(2020) |
| **Cd** | natural metal in water, air, and soil | benign human uterine leiomyoma cells | 10 µM | | 8 weeks | ECM degradation **↑** | uterine fibroids + | Yan Y et al.(2021) |
|  |  | Fibroid cells | 10 µM | | 6 months | heterogeneity ↑ | uterine fibroids + | Yan Y et al.(2022) |
|  |  | Human uterine leiomyoma cells | 0.1, 10µM | | 10 minutes; 24 h | FOXM1/Cyclin D1 **↑** | uterine fibroids + | Yu L et al.(2021) |
| **Cd** |  | Human uterine leiomyoma cells | 0.1,10µM | | 24,48,72 h | GPER/p-src/EGFR/MAPK **↑** | uterine fibroids + | Liu J et al.(2019) |
|  |  | granulosa cells | 0, 0.625, 1.25, 2.5, 5 μM | | 24 h | mitochondrial dysfunction | female infertility | Xu G et al.(2021) |
|  |  | Human:93 female |  | |  | embryo quality **↓** | female infertility **+** | Rodríguez-Díaz R et al.(2023) |
|  |  | Human:890 women with uterine cancer |  | |  | dietary | endometrial cancer | Razumova Z et al.(2022) |
|  |  | breast epithelial cells | 1-3 μM | | 8 weeks | iron concentration **↑**;  ferroportin **↓** | breast cancer **+** | Shan Z et al.(2018) |
|  |  | breast epithelial cells | 1, 3 μM | | 4 weeks | epithelial mesenchymal transition **↑** | breast cancer **+** | Wei Z et al.(2018) |
| **Cd** |  | the ovarian cell line SKOV-3 | 0.1, 1.0, 5.0, 10.0, 50.0 µM | | 24 h; 48 h | P-glycoprotein **↑** | ovarian cancer | Sawicka E et al.(2022) |
|  |  | Human:52 patients with testicular cancer |  | |  | environment | testis cancer **+** | Anđelković M et al.(2023) |
|  |  | Animal: male mice | 2, 4,8 mg/kg, bw | | 8 weeks | LHR; eNOS; 17α-hydroxylase | female infertility | Ren Y et al.(2019) |
| **Lead** | natural metal in water, air, and soil | Animal: female SD rats | 0, 5, 25, 50, 250 mg/L | | 5 weeks | IRE1α-JNK signaling pathway **↑** | female infertility | Shan D et al.(2023) |
| **Hg** | thermometers, light bulbs, and pharmaceutical and beauty products | Human: women aged 20-54 |  | |  | environment | uterine fibroids + endometriosis | Zhang Y et al.(2021) |
|  |  | human endometrial stromal cells | 0, 25, 50, 75, 250, 350 nM | | 24,48,72 h | oxidative stress **↑** | female infertility | Palomar A et al.(2022) |
| **Equol** | the end product of biotransformation of daidzein | Human: women aged 20-54 |  | |  | environment | uterine fibroids **+** | Zhang Y et al.(2021) |
|  |  | Human:1579 women |  | |  | environment | uterine fibroids **+** | Yang F & Chen Y (2023) |
| **Hexavalent chromium** | natural metal in water, air, and soil | Animal: female SD rats | 1, 5 ppm | | postnatal day 22 - 28 | oxidative stress; DNA;  microtubule; chromosomes | female infertility | Wuri L et al.(2023) |
| **Heavy metals** | natural metals in water, air, and soil | Human:838 American women aged 20-44 |  | |  | environment | female infertility **+** | Lin J et al.(2023) |
|  |  | Human:47 women with endometrial cancer and 45 controls |  | |  | environment | endometrial cancer **+** | Atakul T et al.(2020) |
| **Heavy metals** |  | Human:255 males |  | |  | environment | prostate cancer **+** | Lim J T et al.(2019) |
|  |  | Human:162 males |  | |  | environment | prostate cancer **+** | Singh V et al.(2023) |
|  |  | Human:94337 males and females |  | |  | environment | prostate cancer | Cao H M et al.(2023) |
| **Parabens** | antimicrobial preservatives | Human: reproductive-aged Black women |  | |  | environment | uterine fibroids **-** | Wesselink A K et al.(2021) |
|  |  | Human:511 female aged 25-39 |  | |  | antral follicle count **↓** | diminished ovarian reserve **+**  female infertility **+** | Jurewicz J et al.(2020) |
|  |  | Animal: adult mice | 7.5, 90, 450 mg/kg/day | | fetal sex determination period | follicular atresia **↑**  primordial follicles **↓** | diminished ovarian reserve **+** | Li M et al.(2021) |
| **Parabens** |  | Animal: prepubertal mice | 150 μg/g/day, bw | | postnatal day 35-65 | steroidogenesis;  oxidative stress;  reprotoxicity | diminished ovarian reserve **+** | Ara C et al.(2021) |
|  |  | antral follicles of Swiss mice | 0.01-100  μg/mL | | 24-72 h | antral follicle growth;  steroidogenesis | diminished ovarian reserve | Gal A et al.(2019) |
|  |  | Human:101 female |  | |  | environment | diminished ovarian reserve | Pacyga D C et al.(2022a) |
|  |  | Animal: female 8-week-old rats | 100 mg/kg | | 5 weeks | folliculogenesis **↓** | premature ovarian failure **+** | Lee J H et al.(2017) |
|  |  | Animal: female rats | 10,100,1000 mg/kg/day | | gestational 7 -postnatal 22 | steroidogenesis; folliculogenesis | female infertility **+** | Maske P et al.(2018) |
|  |  | Human:1309 women |  | |  | environment | breast cancer **+** | Parada H, Jr. et al.(2019a) |
| **Parabens** |  | human breast tumor tissue |  | |  | environment | breast cancer **+** | Shen X et al.(2018) |
|  |  | human breast tumor tissue |  | |  | environment | breast cancer **+** | Amin M M et al.(2019) |
|  |  | human breast epithelial cells;  BALB/c female mice | 1,5,10,50 uM;  3000,10000μg/kg/day | | 24 h;  4 days | R-loops;  DNA damage | breast cancer **+** | Majhi P D et al.(2020) |
|  |  | Animal: pregnant BALB/c mice | 20,100,10000 µg/kg/day | | pregnancy to lactation | Erα; immune | breast cancer **+** | Mogus J P et al.(2021) |
|  |  | Human:2062 women |  | |  | environment | breast cancer **-** | Wu A H et al.(2021) |
|  |  | Human: 106978 women |  | |  | environment | breast cancer **-** | Rylander C et al.(2019) |
| **Parabens** |  | Human: 46905 women |  | |  | environment | breast cancer **±** | Taylor K W et al.(2018) |
|  |  | Human: woman |  | |  | ER signaling crosstalk | breast cancer **+** | Hager E et al.(2022) |
|  |  | human semen samples | 5, 50, 250, 500,1000 uM | | 0.2, 5 h | reactive oxygen species | male infertility | Samarasinghe S et al.(2018) |
|  |  | Animal: adult female Wistar rats | 100, 200 mg/kg,  bw | | gestation period | mitochondrial bioenergetics | male infertility | Oliveira M M et al.(2020) |
|  |  | Animal: young male Wister rats | 100,200   mg/kg,bw | | 10 days | oxidative stress | male infertility | Martins F C et al.(2023) |
|  |  | Human: 884 pre-conception couples |  | |  | environment | infertility**×** | Ao J et al.(2023) |
| **APEOs** | emulsifiers, detergents, solubilisers, antistatic and dispersing agents | Human:397 women |  | |  | environment | endometrial cancer **+** | Wen H J et al.(2020) |
| **BP-1** | personal care product | human SKOV3 ovarian cancer cells | 10^-9^-10^-6^ M | |  | ERα **↑** Wnt/β-catenin signaling pathways **↑** | ovarian cancer **+** | Liu X et al.(2022) |
| **PFNA** | the manufacture products with water and dirt repellent properties | prostate cancer cells | 0.1- 100uM | | 24,48,72,96 h | environment | prostate cancer **+** | Wei Y et al.(2023) |
|  |  | male human embryonic  stem cells | 2.15,21.5,43  µM | | 10 days | spermatogenesis | male infertility | Steves A N et al.(2018) |
| **Benzene** | volatile organic compounds in petroleum and crude oil products | Human: 1172 men ≤ 75 years of age and 1177 controls |  | |  | environment | prostate cancer **+** | Goldberg M S et al.(2022) |
| **PAH** | products of incomplete combustion such as coal, oil, natural gas, wood, paper | Human:196 girls |  | |  | pubertal milestones | breast cancer | Kehm R D et al.(2021) |
|  |  | normal and breast cancer cells | 6,30,300，16000 nM | | no more than 6 months | antiapoptotic signaling**↑** | breast cancer **+** | Gearhart-Serna L M et al.(2020) |
|  |  | Human: male firefighters in Denmark |  | |  | environment | male infertility | Petersen K U et al.(2019) |
| **EGME** | solvents, antifreeze agents, and coolants in commercial and industrial applications | Animal: male F344/DuCrlCrlj rats | 200, 600, 2000 mg/kg | | 24 h | testicular toxicity | male infertility | Matsuyama T et al.(2018) |
| **Mixtures** | 21 EDCs including parabens, phenols, phthalates and poly-fluoroalkyl substances | Human: 64 women with disease and 86 controls |  | |  | environment | diminished ovarian reserve **+** | Tian T et al.(2023) |
| **Mixtures** | four urinary phenols and eight metabolites of five phthalate diesters | Human:271 women |  | |  | environment | diminished ovarian reserve **×** | Génard-Walton M et al.(2023) |
|  | ethyl paraben, benzophenones, TCS and BPA | Human: U.S. women aged 18-45 |  | |  | environment | female infertility **+** | Arya S et al.(2020) |
|  | DEHP, PFOA,  methylparaben | Human: 333 women in Sweden and Estonia |  | |  | environment | female infertility **+** | Bellavia A et al.(2023) |
|  | DEHP and BPA | Animal: female rats | DEHP:150 mg/kg/day bw;  BPA: 20 mg/kg/day bw | | 30 weeks | Esr1 pathway **↑**  HDAC6 pathway **↑** | breast cancer **+** | Zhang X et al.(2021) |
|  | PCB-153, p,p'-DDE and HCB | Human:330 boys |  | |  | environment | cryptorchidism **×** | Axelsson J et al.(2020) |
| **Mixtures** | p,p'-DDT, p,p'-DDE, 3-PBA or cis-DBCA | Human:68 one-year-old boys |  | |  | environment | hypospadias **×** | Bornman R et al.(2022) |
|  | PFQ and DBP | Animal: pregnant rats |  | |  | androgen signaling pathway | hypospadias **+** | Gray L E et al.(2022) |
|  | dioxins, PCBs and PFAS | Human: Danish and Finnish mother-son |  | |  | environment | hypospadias **×** | Tysman M et al.(2023) |
|  | pesticides, BPA and TCS | Sertoli TM4 cells |  | |  | GJIC, Cx43 and MAPK signaling pathways | testicular cancer **+** | Yawer A et al.(2022) |

**Note:** +: positive ; -: negative;↑: Increase; ↓: Decrease ; ×: unrelated; bw: body weight; h: hour; ER: estrogen receptor.

BPA: Bisphenol A; PAEs: Phthalates; OPEs: Organophosphate esters; PBDEs: polybrominated diphenyl ether; DES: Diethylstilbestrol; HCB: Hexachlorobenzene; PCBs: Polychlorinated biphenyls; TBT: Tributyltin; DDT: Dichlorodiphenyltrichloroethane; DDE: p,p'-dichlorodiphenyldichloroethylene; PFAS: Perfluorinated substances; PFOA: Perfluorooctanoic acid; PFOS: Perfluorooctane sulphonate; PP: Propylparaben; TCS: Triclosan; VCD: 4-vinylcyclohexene diepoxide; TCDD: 2,3,7,8-Tetrachlorodibenzo-p-dioxin; Cd: Cadmium; Hg: Mercury; APEOs: Alkylphenol ethoxylates; BP-1: Benzophenone-1; PBDEs: Polybrominated diphenyl ethers; PFNA: perfluorononanoic acid; PAH: polycyclic aromatic hydrocarbons; EGME: ethylene glycol monomethyl ether.
